# Validation and application of a needs‐based segmentation tool for cross‐country comparisons

**DOI:** 10.1111/1475-6773.13873

**Published:** 2021-11-10

**Authors:** Lize Duminy, Nirmali Ruth Sivapragasam, David Bruce Matchar, Abhijit Visaria, John Pastor Ansah, Carl Rudolf Blankart, Lukas Schoenenberger

**Affiliations:** ^1^ Institute for Health Policy and Health Economics Bern University of Applied Sciences Bern Switzerland; ^2^ Swiss Institute of Translational and Entrepreneurial Medicine Bern Switzerland; ^3^ Program in Health Services and Systems Research Service Duke‐NUS Medical School Singapore Singapore Singapore; ^4^ Duke University Medical Center Duke University Durham North Carolina USA; ^5^ Centre for Ageing Research and Education Duke‐NUS Medical School Singapore Singapore Singapore; ^6^ KPM Center for Public Management University of Bern Bern Switzerland

**Keywords:** access/demand/utilization of services, chronic disease, comparative health systems/international health, geriatrics, health care organizations and systems, integrated delivery systems, modeling: multi‐level, survey research and questionnaire design

## Abstract

**Objective:**

To compare countries' health care needs by segmenting populations into a set of needs‐based health states.

**Data sources:**

We used seven waves of the Survey of Health, Aging and Retirement in Europe (SHARE) panel survey data.

**Study design:**

We developed the Cross‐Country Simple Segmentation Tool (CCSST), a validated clinician‐administered instrument for categorizing older individuals by distinct, homogeneous health and related social service needs. Using clinical indicators, self‐reported physician diagnosis of chronic disease, and performance‐based tests conducted during the survey interview, individuals were assigned to 1–5 global impressions (GI) segments and assessed for having any of the four identifiable complicating factors (CFs). We used Cox proportional hazard models to estimate the risk of mortality by segment. First, we show the segmentation cross‐sectionally to assess cross‐country differences in the fraction of individuals with different levels of medical needs. Second, we compare the differences in the rate at which individuals transition between those levels and death.

**Data collection/extraction methods:**

We segmented 270,208 observations (from Austria, Belgium, Czech Republic, Denmark, France, Germany, Greece, Israel, Italy, the Netherlands, Poland, Spain, Sweden, and Switzerland) from 96,396 individuals into GI and CF categories.

**Principal findings:**

The CCSST is a valid tool for segmenting populations into needs‐based states, showing Switzerland with the lowest fraction of individuals in high medical needs segments, followed by Denmark and Sweden, and Poland with the highest fraction, followed by Italy and Israel. Comparing hazard ratios of transitioning between health states may help identify country‐specific areas for analysis of ecological and cultural risk factors.

**Conclusions:**

The CCSST is an innovative tool for aggregate cross‐country comparisons of both health needs and transitions between them. A cross‐country comparison gives policy makers an effective means of comparing national health system performance and provides targeted guidance on how to identify strategies for curbing the rise of high‐need, high‐cost patients.

**What is known on this topic**

- Population segmentation is a promising approach for health care resource planning and policy making.
- A number of needs‐based segmentation approaches exist, which either are conceptual or use utilization‐based metrics.
- Needs‐based segmentation studies are limited to regional data sources.

**What this study adds**

- The Cross‐Country Simple Segmentation Tool (CCSST) segments individuals into five ordinal medical complexity categories, called “Global Impressions” (GI) segments, and identifies the presence of four patient characteristics, which, if present, would increase the complexity of care, called “Complicating Factors” (CFs).
- The CCSST is applied to the SHARE panel survey dataset and allows for comparisons across countries.
- Our approach helps policy makers identify country‐specific areas of analysis of services and factors that may confound transition rates.

## 1 INTRODUCTION

Chronic conditions have been called “the healthcare challenge of this century” by the World Health Organization.^1^ The prevalence of patients with numerous and complex health care needs related to one or more chronic conditions is expected to increase as life expectancy continues to rise.^2^, ^3^ An increasing life expectancy also exposes individuals to a greater number of changes over their life course, particularly to older‐age life transitions not captured in their medical diagnoses. Changes over the life course, such as changes in social networks, relationships, living arrangements, or employment, influence the complexity of individuals' health care, including their ability to meet their own basic needs and rely on support from others.^4^ Therefore, the health system is facing not only an increase in the number of individuals with specific clinical diagnoses^3^ but also a growing number of individuals with complex needs related to a combination of multiple medical conditions and health‐related social needs.

Lynn et al.^5^ developed a needs‐based population segmentation approach for improving a health system's effectiveness and efficiency in meeting the needs of the population it serves. Within a population, this approach identifies segments or clusters of individuals with similar care goals and similar types and intensity of needs. Such an ontology can, for example, measure the extent to which a particular population segment has less than desirable outcomes relative to a given benchmark. Policy makers can use these estimates to assess whether that segment's health and health‐related social service needs are met and, if not, to develop targeted interventions.^5^

While no clear definition of a “need” exists, a service is defined as “needed” when a typical individual with a set of characteristics that define a segment will likely benefit from receiving that service (in terms of reducing the likelihood of experiencing a more adverse health state).^6^ To date, the operationalization of population needs‐based segmentation has been restricted to specific subgroups (e.g., the frail elderly or individuals with possible palliative care needs)^7^, ^8^, ^9^, ^10^ or to data‐driven approaches relying on electronic records to cluster individuals according to the risk of both poor outcomes and high cost.^8^

In this paper, we introduce an adapted version of the *Simple Segmentation Tool (SST)* ^11^—developed by Duke‐NUS Medical School—that we call the “Cross‐Country Simple Segmentation Tool” (CCSST). The CCSST segments the population into clinically significant global impressions (GI) segments (i.e., categories in terms of health status and medical complexity) and complicating factors (CFs) (e.g., experiencing fragmented care) are indicative of health and health‐related social services needs, respectively. Operationally, for the needs‐based population segmentation approach, a consensus panel of medical and social service experts defined “need” as any actionable service with a high probability of notable benefit.^7^

In our framework, health needs are principally services that would need to be provided by a physician or other health provider trained and licensed to diagnose, prescribe, and perform procedures; and health‐related social services needs are those that a physician, nurse, social worker, family member, volunteer, or even a specific technology can provide. While the SST was originally developed for clinical settings, it was modified for administration through special‐purpose community surveys,^11^, ^12^ from which the CCSST is adapted. The CCSST uses data from the Survey of Health, Aging and Retirement in Europe (SHARE) and related international surveys available from the Gateway to Global Aging Data.^13^ Using data that are readily available in many countries allows us to compare health care needs across countries.

The objective of this article is to present the CCSST and demonstrate a cross‐country benchmarking application, comparing countries according to (1) the fraction of individuals with high medical needs and (2) the relative hazards of transitioning between lower and higher needs segments, and between those segments and death.

## 2 METHODS

The methods section is structured as follows. First, we briefly introduce the survey data. Second, we present and describe the CCSST, a segmentation tool comprising five ordinal GI segments and four binary CF indicators. The GI segment categorizes individuals into one of five ordinal medical complexity categories. The four CFs identify patient characteristics that, if present, would increase the complexity of care for the conditions in the GI designation. Third, using Cox proportional hazard models, we assess the CCSST for predictive validity by modeling the association of the CCSST segments with mortality. Fourth, we describe how we applied the CCSST to cross‐country comparisons by (a) comparing the proportion of individuals in different states of need and (b) comparing proportional hazards of transitioning between those states using a continuous‐time Markov process.

### 2.1 Description of survey data

The SHARE is a multidisciplinary, cross‐national panel database consisting of seven biennial survey waves. It contains microdata on health, socioeconomic status, and the social networks of individuals older than 50 years.^10^ The survey was conducted biannually in 29 countries, starting in 2004. Interviews were conducted in person with the target individuals and their partners. To ensure reliable convergence of the transition probability estimation, we include those 14 countries with at least 8 years of survey participation. The 14 countries are Austria, Belgium, Czech Republic, Denmark, France, Germany, Greece, Israel, Italy, the Netherlands, Poland, Spain, Sweden, and Switzerland.

### 2.2 Development of the CCSST: Mapping survey data to GI and CFs

The CCSST is based on both clinical and survey versions of the SST, which has been validated for inter‐rater reliability in a clinical setting,^7^ and for predictive validity in both clinical and survey settings^11^, ^12^ as well as having face validity.^7^ In both of these applications, questions were added to the data collection instruments, with the specific goal of assessing a specific set of GI categories and CFs. However, given that the survey data were not created for this purpose, we designed and constructed a mapping algorithm by using the foundational principles of the SST (see Supporting Information Data S1 for the original SST). We found the data sufficient for the five ordinal GI segments: healthy, chronic asymptomatic, chronic symptomatic, long course of decline, and limited reserve with serious exacerbation—in line with the previous survey version of the SST.^12^ Although the original SST included a sixth state (short period of decline before dying), predicting rapid decline with survey data is not feasible. We, therefore, collapsed the sixth state, short period of decline before dying, into the fifth state, limited reserve, and serious exacerbation.^12^ The qualifying criteria for each GI segment are defined through clinical indicators, self‐reported physician diagnosis of chronic disease, and performance‐based tests conducted during the survey interview.

A trade‐off to limiting ourselves to data in aging surveys,^13^, ^14^ thereby making the tool widely accessible, was that the CCSST was able to capture only four of the eight CFs described in the original SST: functional assessment (dependence on caregiver assistance), social support in case of need, frequent transitions between inpatient and outpatient care, and needing to take five or more prescription medications daily. The other CFs are need for nursing or rehabilitation services, activation in care, disruptive behavioral features, and having multiple health care providers in multiple locations. Even though we were able to capture only four CFs, preliminary results on the effect of the four we identified had been found earlier to have a significant association with adverse health outcomes.^11^ We confirmed the consistency of all variables used for both GI segments and CFs across all waves for all countries. The descriptive mapping file for the GI segments and CFs is given in Table 1.

**TABLE 1:** Descriptive mapping table for GI segments and CFs

GI segments^a^	CCSST definition	Example	Segmentation condition	
Healthy	No more than minimal symptomatic conditions and no asymptomatic conditions that increase risk	Acute URTI	No Chronic disease, no difficulty with any activity of daily living, not cognitively impaired and not classified as frail	
Chronic asymptomatic	Chronic conditions *(not curable once acquired or has persisted >3 months despite treatment)* that are asymptomatic but notable for increasing preventable risk	Asymptomatic diabetes	EITHER	Non‐life‐threatening chronic disease
				AND Global Activity Limitation Index indicated “not limited”
			OR	Classified as depressed according to the EURO‐D scale
				AND Global Activity Limitation Index indicated “not limited”
Chronic stable	*Chronic conditions that are relatively stable* but associated with symptoms that interfere with or restrict usual function or would generally be sufficient to trigger care‐seeking. Include conditions that are *silent (relatively asymptomatic) but severe*	Symptomatic Parkinson's disease	EITHER	Life‐threatening chronic condition
				AND Global activity limitation index indicated either “not limited” or “limited, but not severely”
			OR	Non‐life‐threatening chronic disease
				AND Global Activity Limitation Index indicated either “limited but not severely” or “severely limited”
			OR	Classified as depressed according to the EURO‐D scale
				AND Global activity limitation index indicated either “limited but not severely” or “severely limited”
Long course of decline	*Long* (*months to years*) dwindling course of loss of reserve in multiple organ systems; typically elderly. *Decline* may be characterized by geriatric syndromes or recurrent exacerbations of multiple co‐dominant medical (nonsocial) conditions	Frail elderly with dementia	Classified as frail	
			OR Classified as cognitively impaired	
Limited reserve and serious exacerbation	*Single dominant* medical (nonsocial) condition associated with *recurrent exacerbations*	Frequent flares of COPD	Life‐threatening chronic condition	
			AND Global Activity Limitation Index indicated “severely limited”	

CFs	CCSST definition	Example	Segmentation condition
Functional assessment	Deficit implies dependence on caregiver assistance to perform basic or instrumental activities of daily living: The individual is otherwise unable to perform tasks independently	0 = no deficit 1 = any ADL or IADL deficit	*Basic activities of daily living*: Dressing (including putting on shoes and socks), walking across a room, bathing or showering, eating (including cutting up food), getting in or out of bed and using the toilet (including getting up or down) *Instrumental activities of daily living*: Using a map, preparing a hot meal, shopping for groceries, making a telephone call, taking medications, doing work around the house or garden, and managing money (paying bills, keeping track of expenses)
Social support in case of need	Family or friends who provide support through companionship and basic health care services in case of need. Companionship support includes assistance with major medical decision making. Basic health care services include support with activities of daily living/instrumental activities of daily living, as well as skilled nursing tasks (i.e., health care tasks requiring specific skills training. Can often be performed by a caregiver or domestic worker if properly trained).	0 = has support for basic health care services or companionship 1 = no support	*Support for basic health care services or companionship*: Someone is in the household helping regularly with personal care. Any friend, neighbor, or family member from outside the household has provided any kind of help in the past 12 months A family respondent is helping with the completion of the survey
Frequent transitions between inpatient and outpatient care	Number of overnight inpatient admissions during the past 12 months. Individuals with a high number of transitions between care venues are more likely to experience fragmented care.	0 = less than three times 1 = three or more times	*Individuals most frequently transitioning between venues of care*: Using information regarding the number of times stayed overnight in hospital during last 12 months. Create a threshold identifying the individuals who are in the top 95% of respondents, based on the sample in wave 1: three times
Polypharmacy (five or more medications prescribed per day)	The burden of having five or more prescription medications to take daily. Prescription medication categories are counted.	0 = fewer than five prescription medication categories 1 = five or more prescription categories	*Prescription medication*: Count the number of categories of medications reported taking. Categories include drugs for: high blood cholesterol, high blood pressure, coronary or cerebrovascular diseases, other heart diseases, diabetes, joint pain or for joint inflammation, other pain (e.g., headache, back pain, etc.), sleep problems, anxiety or depression, osteoporosis, stomach burns, bronchitis, and suppressing inflammation (only glucocorticoids or steroids)

Abbreviations: CCSST, cross‐country simple segmentation tool; CF, complicating factors; GI, global impressions.

^**a**^ GI segments become consecutively more severe. If an individual qualifies for more than one health state, that individual should be assigned to the most severe of the health states.

The clinical indicators we used for segmentation include frailty, the global activity limitation index, activities of daily living, the EURO‐D depression score, and cognitive impairment. Frailty was determined through the SHARE operationalization^15^ of Fried's “Frailty as a phenotype.”^16^ The global activity limitation index is a global single‐item instrument that measures long‐standing activity limitations (6 months or more) that general health problems cause and that inhibit activities commonly undertaken by survey participants.^17^, ^18^ The EURO‐D depression symptom scale is a 12‐point index identifying an individual's validated depression state for comparing the prevalence and risk of depression across countries.^19^, ^20^, ^21^ Finally, we measure cognitive impairment by individuals' scoring below a cutoff value in a performance‐based cognitive function score consisting of the mean normalized score of five cognitive function tests: verbal fluency, immediate recall, delayed recall, orientation, and numeracy.^22^ The cutoff value was defined as 1.5 standard deviations below the mean score obtained by the total survey Wave 1 sample.

Chronic disease diagnoses—classified as either potentially life‐threatening or non‐life‐threatening—were determined through discussions with physicians on the project team and individuals involved in designing previous versions of the SST.^7^, ^11^, ^12^ As chronic conditions are by definition persistent over the life span, all participants, from the time of their diagnosis, are assigned to all future waves. Life‐threatening chronic diseases were defined as conditions likely to dominate an individual's health care management and planning—including exacerbations not dealt with by a primary physician, and in which the likely effects of failure to monitor physiological parameters with prompt follow‐up would be serious (e.g., avoidable hospitalization or death). The self‐reported physician‐diagnosed conditions identified as life‐threatening chronic diseases are myocardial infarction, congestive heart failure, stroke, chronic obstructive lung disease, hip fractures, and cancer (including leukemia or lymphoma, but excluding minor skin cancers). Hip fractures are considered life‐threatening across all health systems because the morbidity of health conditions is assessed independently of available health system resources.^23^

Non‐life‐threatening chronic diseases were defined as all other conditions requiring continuous medical surveillance after symptoms have persisted for more than 3 months. Medical surveillance of non‐life‐threatening chronic diseases can usually be conducted by a combination of the individual, their primary care provider, and social care actors within the community. The following self‐reported diagnoses were identified as non‐life‐threatening chronic diseases: high blood pressure or hypertension, high blood cholesterol, diabetes or high blood sugar, stomach or duodenal ulcer, peptic ulcer, Parkinson's disease, cataracts, and arthritis‐related conditions. For arthritis‐related conditions, a composite indicator combines the diagnoses osteoarthritis, rheumatism, and osteoporosis available only in the first four waves of the survey, with rheumatoid arthritis and osteoarthritis or other rheumatism available from the fifth wave onward. All self‐reported diagnoses or composite indicators used are present in each wave and show consistency across waves.

The CFs were selected by a consensus panel of medical and social service experts from the evidence that such factors were predictive of adverse outcomes and that receiving the corresponding non‐physician and social services has the potential for alleviating the CF effects.^7^ The list did not include social determinants of health, such as housing or food insecurity, that are outside the control of a conventional health system.

### 2.3 Assessing the predictive validity of the CCSST

To assess the predictive validity of the CCSST, we assigned individuals to segments by GI and the presence of at least one CF. In the absence of data on the utilization of acute services (e.g., emergency department visits, hospitalization, or nursing home placement), we assessed predictive validity by using the degree to which baseline categorization was associated with mortality. Using Cox proportional hazard modeling, we calculated hazard ratios for mortality between least and most severe GI categories (healthy and limited reserve and serious exacerbation), and between individuals with a CF and individuals without a CF, for each of the four variables measured, while controlling for age, age‐squared, and gender.

### 2.4 Cross‐country ranking of per capita high medical needs

For our results to match the national populations of individuals who are 50 years or older, we applied calibrated cross‐sectional weights computed separately by country^16^, ^17^ to individuals segmented into GI segments, with and without CFs to each wave. The weighted segments were grouped into those indicative of high medical needs GI segments (long course of decline or limited reserve and serious exacerbation) and those who were not (healthy, chronic asymptomatic, or chronic symptomatic). The results are ranked according to the proportion of each population with high medical needs.

### 2.5 Cross‐country comparison of hazard of transition

Once again, individuals were grouped into those who had high medical needs (i.e., into one of the two most severe GI segments) and those who did not. Transition probabilities were estimated on the assumption that the trajectories of individuals in panel data reflect a continuous‐time Markov process.^24^, ^25^ This approach incorporates the available, incomplete data on transitions from one state to another, with the instantaneous state determined during the non‐informative biannual panel interview wave across interview waves.

This approach allows for multiple observation types: two health states, a censored state determined at arbitrary times, and death (which is determined either exactly or arbitrarily). Censored observations are observations in which individuals had some form of personal interaction with representatives of the survey but did not provide sufficient information for us to segment them by using the CCSST. As these individuals were confirmed to be alive, they were assumed to either have high medical needs or not. Instantaneous transitions,^26^ governed by a set of transition intensities, were permitted in both directions—between not having high medical needs and having high medical needs, with death defined as an absorbing state, that is, allowing transitions only into the death state. We modeled the instantaneous risk of moving from one state to another by using log‐linear models that control for country, gender, age classes (50–59 years, 60–69 years, 70–79 years, 80+ years), and the presence of any CF as covariates.^27^ Estimates from these log‐linear models are exponentiated and interpreted as hazard ratios.

We ensured a reliable model convergence by (a) minimizing the number of groups (high medical needs, no high medical needs, and death) by including CFs as a time‐inhomogenous covariate, rather than as part of the state stratification, and (b) restricting our analyses to countries with at least 8 years of observations to ensure that enough state transitions occurred. We evaluated different combinations of GI segments per health state to make certain that the rankings were robust and not the result of modeling Simpson's paradox, in which a trend appears in several different groups of data but disappears or reverses when these groups are combined.^9^ We performed the analyses in the R statistical computing environment^10^ by using the msm software package.^11^ Although we estimate transition probabilities using a continuous‐time Markov process, a Markov simulation model is beyond the scope of this paper.

## 3 RESULTS

### 3.1 Descriptive statistics of CCSST

For our 14 countries in the survey dataset between 2004 and 2017, 96,396 individuals contributed 270,208 observations. These observations included all regular panel interviews (232,386 observations), life‐course interviews (27,617 observations), and end‐of‐life interviews (10,205 observations) conducted with the next‐of‐kin in the case of death. Of the 232,386 regular panel interview observations, the CCSST algorithm was able to assign 229,722 (98.9%) into a GI category (ranging from 97.6% for Austria to 99.5% for Denmark). A similarly high proportion of observations in the population were assigned to the presence or absence of a CF (231,953 observations, 99.8%), ranging from 99.5% for Israel and 99.9% for Switzerland. Among the survey population included, 10,205 individuals were confirmed deceased during the follow‐up. See Supporting Information Data S2 for the segmentation results, shown per country, per interview wave.

### 3.2 Predictive validity of the CCSST

**FIGURE 1:**
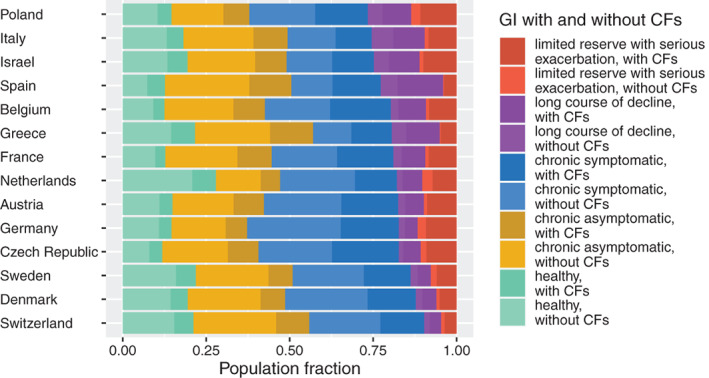
Fractional CCSST‐assigned global impressions (GI) segments with and without complicating factors (CFs) per country, ranked by the population fraction with high medical needs (i.e., the two most severe GI segments). Data were collected in 2015 for all countries except the Netherlands, for which we use data from 2013 [Color figure can be viewed at wileyonlinelibrary.com]

**FIGURE 2:**
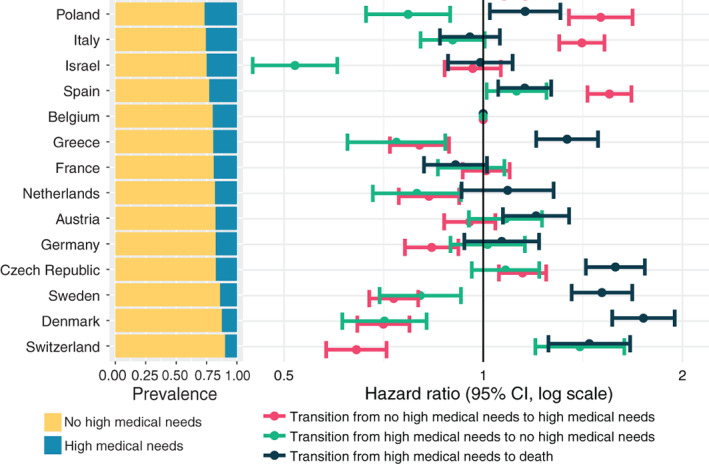
Cross‐country comparison of (1) prevalence of having high medical needs (i.e., classified in either of the two most severe global impressions (GI) segments) or not, and (2) hazard rates between no high medical needs and high medical needs segments, and between high medical needs and death [Color figure can be viewed at wileyonlinelibrary.com]

In the Cox regression adjusted for age, age‐squared, and gender, hazard ratios for mortality associated with high medical needs were significantly greater than 1 for all countries (see results on the predictive validity of GI segments in Table 2). Similarly, having a CF was associated with a higher likelihood of mortality, with 12 of 14 countries having hazard ratios statistically significant at the 1% level (see results on the predictive validity of CF segments in Table 2). The Kaplan–Meier survival curves associated with mortality by GI segments and the presence of any CFs appear in Supporting Information Data S3 (Figures 1A–D and 2A–D).

**TABLE 2:** Predictive validity of the GI segments and presence of CFs

CCSST aspect	Country	Observations	Deaths	Cox regression of time to death controlled for gender age, age‐squared			
				Hazard ratio	*p* value	CI – LB	CI – UB
GI segments	Austria	5162	565	4.39	<0.001	3.27	5.88
	Germany	5864	392	6.27	<0.001	4.34	9.06
	Sweden	5289	727	5.29	<0.001	4.03	6.94
	Netherlands	3255	265	4.86	<0.001	3.28	7.19
	Spain	7055	1299	3.76	<0.001	2.89	4.88
	Italy	5304	714	3.68	<0.001	2.72	4.98
	France	6092	634	4.61	<0.001	3.31	6.41
	Denmark	4651	629	4.83	<0.001	3.63	6.42
	Greece	3662	582	1.92	<0.001	1.37	2.69
	Switzerland	3630	286	3.41	<0.001	2.25	5.18
	Belgium	6772	701	5.24	<0.001	3.89	7.07
	Israel	3035	561	4.00	<0.001	2.83	5.66
	Czech Republic	6391	952	4.08	<0.001	3.15	5.28
Presence of CFs	Poland	2016	504	2.23	<0.001	1.58	3.15
	Austria	5305	581	2.14	<0.001	1.80	2.55
	Germany	5929	398	2.12	<0.001	1.71	2.62
	Sweden	5346	739	1.74	<0.001	1.49	2.02
	Netherlands	3557	275	1.90	<0.001	1.48	2.43
	Spain	7144	1312	1.50	<0.001	1.33	1.69
	Italy	5396	720	1.49	<0.001	1.28	1.75
	France	6227	649	1.82	<0.001	1.54	2.16
	Denmark	4674	635	1.76	<0.001	1.49	2.06
	Greece	3737	597	1.18	0.057	1.00	1.41
	Switzerland	3672	291	1.71	<0.001	1.35	2.16
	Belgium	6814	706	1.70	<0.001	1.45	2.00
	Israel	3115	572	1.76	<0.001	1.46	2.12
	Czech Republic	6457	962	1.76	<0.001	1.54	2.01
	Poland	2026	506	1.18	0.094	0.97	1.43

Abbreviations: CCSST, cross‐country simple segmentation tool; CF, complicating factors; GI, global impressions; LB, lower bound; UB, upper bound.

### 3.3 Cross‐country ranking of per capita high medical needs

The most recent country‐specific per capita proportions of GI segments with and without CFs are shown in Figure 1. For all countries except the Netherlands, the most recently available wave is Wave 6, collected in 2015. The most recent regular Dutch survey wave available is Wave 5, collected in 2013. The countries are ranked by the relative size of their populations that are in either of the two most severe GI segments: long course of decline or limited reserve with serious exacerbation. Individuals in either of these GI segments have high medical needs. Switzerland has the lowest fraction of individuals in high medical needs segments, followed by Denmark and Sweden. At the other end of the spectrum, Poland has the highest fraction of individuals in high medical needs segments, followed by Italy and Israel. The weighted country‐specific per capita proportions of each segment appear in Supporting Information Data S4.

### 3.4 Cross‐country comparison of hazard of transition

We compare disease progression and recovery rates between countries using hazard ratios shown in Figure 2. First, the hazard ratio for transitioning from medically severe to death is generally inversely proportional to the fraction of the population in the high medical needs segment (i.e., the fewer individuals with high needs, the more likely they are to die). Second, with exceptions (e.g., Germany, Israel, Poland, and Switzerland), the relative hazard for moving between high and low medical needs categories is similar (i.e., the hazard of developing high needs is similar to the hazard of moving to low needs). Third, for the countries with the lowest fraction of high medical needs individuals, not only is the hazard of transition from high needs to death higher, the hazard of transitioning from low to high medical needs is lower (i.e., for countries with the lowest fraction of individuals with high medical needs, the risk of entering the high medical needs category is lower, and the risk of exiting via death is higher).

Our results use 95% confidence intervals for the hazard ratios to determine whether differences or similarities in mean values are due to chance. The non‐overlapping confidence intervals in Figure 2 suggest differences across countries that are likely not due to simple random effects. See Supporting Information Data S5 for all hazard ratios for transition—including those for model covariates gender, age classes (50–59 years, 60–69 years, 70–79 years, 80+ years), and the presence of any CFs.

## 4 DISCUSSION

This paper presents a method for segmenting populations according to features that relate to actionable health needs. Operationalized as the CCSST, this method can be applied to internationally available, nationally representative datasets. We further illustrate the application of the CCSST to the survey data from 14 countries. In addition to having clinical face validity (i.e., corresponding to how health and social service providers generally classify patients with similar needs), the GI and CF categories of our CCSST application are predictive of mortality. Moreover, we introduce and demonstrate two valuable cross‐country analyses of the CCSST classification. First, to assess cross‐country differences in the fraction of individuals with different levels of medical needs, we show the segmentation cross‐sectionally. Second, we compare the differences in the rate at which individuals transition between levels of medical needs and death.

Rapid population aging and accompanying increases in longevity have highlighted the fragmentation of greater health care service provision in many developed and developing countries. As health care systems are in a period of rapid change from acute to chronic service delivery, policy makers need new performance metrics to help them learn which strategies are successful. As the CCSST allows for international comparison, it has great potential for supporting support policy makers in achieving their goals.

Unlike population segmentation approaches that focus on predicting poor outcomes, utilization, or cost, the CCSST methodology is explicitly linked to typical, actionable needs. Since the GI segments were defined by clinicians as meaningfully different in terms of type and intensity of medical service needs,^7^ any transition across GI segments represents a clinically significant change to an individual's health. Therefore, by extension, any transitions between collapsed categories indicate a clinically significant change in an individual's health.

Analyzing transition rates, therefore, helps policy makers think about and determine how undesirable transitions across health states—high rates of transition from low needs to high needs and high needs to death, or low rates of transition from high needs to low needs—might be handled at a whole‐system level. For example, high rates of transition to high needs segments could be the result of unaddressed risk factors (e.g., smoking, excessive alcohol use, untreated diabetes, low rates of either vaccination for preventable conditions or screening for major treatable ones). High rates of mortality among individuals with high medical needs could be attributed to problems with the accessibility and affordability of medical services or lack of attention to CFs, thereby making medical services more difficult and less effective. In short, these metrics provide a guide to better assessing where the health system is less than effective in meeting health and health‐related social service needs, in turn directly leading to interventions aimed at efficiently meeting those needs.

While we propose that applying a needs‐based segmentation measure such as the CCSST can contribute to positive action, we recognize that, in isolation, neither comparisons between the proportion of the population within each health state nor the differences in proportional hazards of transitions can be directly used for assessing the quality of health system performance. Health outcomes reflect a myriad of causes, some of which may not be within the resources or ambit of the health system. Nevertheless, our needs‐based segmentation approach can help identify ecological factors, such as social determinants of health (e.g., income, housing, pollution), thereby allowing health system leadership to provide critical input to broader public policy making.

### 4.1 Strengths and limitations of the study

This study has several major strengths. First, the CCSST is based on a conceptual framework that explicitly links the features of individuals to needs that, if met, have the potential for improving health. Second, the approach provides an opportunity for evaluating and comparing needs‐based population segments across time, across jurisdictions, thanks to the availability of compatible data collection efforts. The CCSST can be applied to most countries that conduct a health and aging survey, thereby allowing a form of benchmarking across countries and health systems in a period when dynamic complexity in health care service provision is rapidly increasing. Third, while the CCSST may lose some of the classification precision of a clinician's assessment, the CCSST has the advantage of not requiring information that depends heavily on health service utilization. It focuses instead on the self‐report of health conditions and the impact of those conditions on perceived well‐being.

Indeed, segmenting the population without relying on utilization data constitutes an advantage over the original survey version of the SST. While the original survey version of the SST was designed for mapping the clinician‐completed version, it uses emergency room hospitalizations in assigning segments.^12^ This feature of the CCSST will be incorporated into future survey versions of the SST.

One limitation of this study is that the segmentation is not necessarily equally accurate from country to country, because the comparisons presume minimal differences in, for example, cultural variations in how people answer specific questions. Chronic conditions most likely to be affected by cultural variation are cognitive impairment and depression. However, our study partly mitigates the variation in accuracy by applying performance‐based tests and validated scales for cognitive impairment and depression, respectively. Moreover, one of the two most severe GI segments, long course of decline, is defined independently of self‐reported physician diagnoses. The hazard ratios for mortality from the Cox proportional hazard models show similar ordering in the degree of severity of GI segments. This ordering suggests (a) similar predictive validity for all countries and (b) the absence of major misclassification bias across countries.

The final limitation is that cross‐country comparisons of the distribution of individuals across health states may be biased if the distribution of health states is not representative of the overall country distribution of the health state. We mitigate this limitation by relying on transition probabilities estimated by using observed transitions across health states, not on health state prevalence. Thus, local datasets with overrepresentation of certain health states can still yield valid transition probabilities under the assumption that individuals in overrepresented health states are still representative of the local population in terms of their transition rates.

### 4.2 Implications

Future applications of this work include country‐specific projections of the proportion of the future health status by using simulation modeling^12^ to examine the potential impact of various policy actions aimed at more effectively meeting population needs.^28^ Country‐specific modeling applications could potentially increase their number of states to incorporate CFs into their state stratification, because a larger number of states would reliably converge without the computational complexity of a 14‐level country covariate. Other applications include determining the overall health system performance across countries through a utility‐based evaluation of the CCSST health states. The first step would be to transform the SHARE quality of life indicator, the CASP‐19,^13^ into cardinal utilities through, for example, discrete choice experiments.^14^

Further research is also needed for comparing the degree of bias present in the segmentation across countries. Future studies should compare the assessments resulting from the application of the CCSST to either (a) the assessments of an international group of clinicians or (b) entirely objective measures such as biomarkers, potentially including analyses of dried blood spot assays.^29^ Additional further research includes applying the CCSST to related international surveys,^13^ including the US Health and Retirement Study, to accelerate a deeper understanding needed to improve population health globally.

## 5 CONCLUSIONS

The CCSST is an innovative tool for aggregate cross‐country comparisons of both health needs and transitions between them. A cross‐country comparison gives policy makers an effective means of benchmarking national health system performance and provides targeted guidance on how to identify strategies for curbing the rise of high‐need, high‐cost patients and generally promoting coherent efforts at system improvement.

## Supporting information


**Data S1.** Supporting information.

**Data S2.** Supporting information.

**Data S3.** Supporting information.

**Data S4.** Supporting information.

**Data S5.** Supporting information.
