# Supplementary material for: Validation and application of a needs‐based segmentation tool for cross‐country comparisons
Source: Health Serv Res. 2021 Nov 10;56(Suppl 3):1394–404. doi: 10.1111/1475-6773.13873 (PMC8579203; doi:10.1111/1475-6773.13873)
Supplement: Supplementary file 1 — Data S1. Supporting information. [file HESR-56-1394-s005.pdf]

Simple Segmentation Tool (SST) V3 **16 June 2017**

Patient initials: ____________________ **Sex:** ______________ **Age:** ________ **Ward / room / bed number:** ____ /____ /_____

Evaluator name: _________________________ **Date of evaluation:** ________________________ **Study ID:** _______________

Global impression of patient (Circle (O))

- I — Healthy
- II — Chronic condition(s), asymptomatic
- III — Chronic condition(s), stable but moderately/ seriously symptomatic or silently severe
- IV — Long course of decline
- V — Limited reserve and serious exacerbations
- VI — Short period of decline before dying

Complicating factors	Level (Circle (O))		
**A. Functional assessment, ADL/IADL**<br>0 = no deficit<br>1 = any IADL deficit, no ADL deficit<br>2 = any ADL deficit	0	1	2
**B. Nursing type skilled task needs**<br>0 = none<br>1 = moderate (1 task)<br>2 = high (2 or more tasks)	0	1	2
**C. Rehabilitation type skilled task needs**<br>0 = none<br>1 = moderate (1 task)<br>2 = high (2 or more tasks)	0	1	2
**D. Organization of care**<br>0 = patient will see no more than 1 doctor, from 1 site of care<br>1 = patient will see more than 1 doctor, from 1 site of care<br>2 = patient will see more than 1 doctor, from more than 1 site of care	0	1	2
**E. Activation in own care**<br>0 = ready, understands and interested in treatment; active cooperation and participative<br>1 = unsure but willing to cooperate, can be expected to provide at least a moderate level of self-care<br>2 = major disconnect, unaware/ no insight, may be defiant and can't be expected to provide even a modest level of self-care	0	1	2
**F. Disruptive behavioral/psychiatric issues**<br>0 = none<br>1 = 1 or more, not significantly affecting care<br>2 = 1 or more, significantly affecting care	0	1	2
**G. Social support in case of need**<br>0 = has support for both basic healthcare services and companionship<br>1 = no support for either basic healthcare services or companionship<br>2 = dysfunctional social circumstance	0	1	2
**H. Hospital admissions in last 6 months**<br>0 = none<br>1 = 1 to 2<br>2 = 3 or more	0	1	2
**I. Polypharmacy**<br>0 = fewer than 5 prescription medications<br>1 = 5 to 8 prescription medications<br>2 = 9 or more prescription medications	0	1	2

© 2017 Duke-NUS Medical School, NUS
(Produced by David Bruce Matchar, Chong Jia Loon, Rita Sim Siew Choo)

Guidelines: Circle only one Global Impression category most likely to determine health needs over the next months to years for the most severe active condition. Complicating factors are rated based on current condition unless otherwise indicated. If the likelihood is 50/50 between 2 categories, assume higher severity category.

Global impression	Definition	Example
I. Healthy	No more than minimal symptomatic conditions and no asymptomatic conditions that increase risk.	Acute URTI
II. Chronic conditions, asymptomatic	Chronic conditions (**not curable once acquired or has persisted >3 months despite treatment**) that are asymptomatic, but notable for increasing preventable risk. **(If chronic condition-related symptoms that interfere with/restrict normal function are present (at time of assessment), or if unclear whether acute symptoms are chronic condition-related, assign to Category III**).	Asymptomatic diabetes
III. Chronic conditions, stable but moderately/ seriously symptomatic or silently severe	Chronic conditions that are relatively stable, but are associated with symptoms that interfere with/restrict usual function or would generally be sufficient to trigger care seeking (e.g., pain, fatigue). Also, include conditions that are **silent (relatively asymptomatic) but severe** (e.g., end stage renal failure, malignancy).	Symptomatic Parkinson's disease
IV. Long course of decline	Long (**months to years**) dwindling course of loss of reserve in multiple organ systems; typically elderly. **Decline** may be characterized by geriatric syndromes (e.g., *recurrent falls/gait instability, urinary/bowel incontinence, sleep disorders, poor feeding/malnutrition/inanition, fainting/syncope, dizziness, iatrogenic disorders, pressure ulcers, elder mistreatment, constipation, cognitive impairment [includes dementia, depression, delirium (ever)], frailty [includes decline in functional reserves]*) or **recurrent exacerbations** for multiple co-dominant medical (nonsocial) conditions.	Frail elderly with dementia
V. Limited reserve and serious exacerbations	**Single** dominant medical (nonsocial) condition associated with **recurrent exacerbations.** (**Recurrent exacerbations** are defined as 3 or more healthcare episodes (e.g., emergency department visits, unplanned hospital admissions, urgent primary care attention) in prior 6 months for the condition, may **include** episode at time of assessment).	Frequent flares of COPD
VI. Short period of decline before dying	Severe life limiting conditions **typically** for **end-of-life type palliative management** (palliative management is not the same as conservative or 'max ward' management).	Advanced cancer

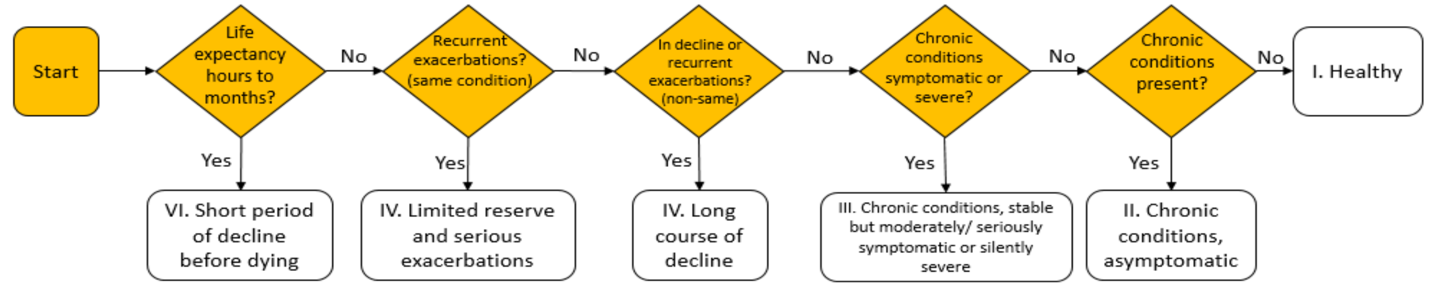

Complicating factors	Level and legends
**A. Functional assessment**<br>(Definition: Deficit implies dependence on caregiver assistance to perform basic or instrumental ADL; Patient is unable to perform tasks independently otherwise.)	**Basic ADLs:** Bathing Dressing Toileting Transferring Continence Feeding<br>**Instrumental ADLs:** Ability to use telephone Shopping Food preparation Housekeeping Laundry Use of transportation Responsibility for own medication Ability to handle finances
**B. Nursing type skilled task needs**<br>**C. Rehabilitation type skilled task needs**<br>(Definition: Healthcare tasks which require specific skills training to perform. Often can be performed by patient/caregiver/domestic worker if properly trained.)	**Nursing type tasks:**<br>Wound dressing<br>Injections<br>Change of feeding tube<br>Urinary catheter<br>**Rehabilitation type tasks:**<br>Physiotherapy<br>Speech therapy<br>Occupational therapy
**D. Organization of care**<br>(Definition: Degree of care fragmentation due to high number of doctors and institutions involved in patient care.)	Sites of care can include primary care clinics, hospitals, nursing home, home, etc.
**E. Activation in own care**<br>(Definition: Knowledge, skill, and confidence for managing one's health and healthcare as well as cooperation with treatment.)	Cooperation with treatment includes at least a modest degree of self-care consistent with functional status (e.g., consistently takes prescribed medications).
**F. Disruptive behavioural issues**<br>(Definition: Disruptive behavioural issues requiring support for caregiver.)	Behavioral issues are disruptive if they **significantly affect care; which is defined as typically requiring time and attention from people in patient's environment,** or inducing distress.<br>Substance abuse Self-harm Confusion/forgetfulness<br>Depression Dissociative problems Abusiveness<br>Anxiety Night-time wandering Physical aggression towards others
**G. Social support in case of need**<br>(Definition: Family or friends who provide support through companionship and basic healthcare services in case of need.)	Companionship support includes aid with major medical decision making. Basic healthcare services include support with ADL/IADL as well as skilled nursing type tasks (see Complicating Factor C above). Dysfunctional social circumstance is typically characterized by instances of conflict, neglect or abuse within patient's family which significantly affects care.
**H. Hospital admissions in last 6 months**<br>(Definition: Overnight inpatient hospital ward stays for diagnosis and treatment.)	Does not include any hospital admissions associated with the present medical presentation. Also exclude community hospital admissions.
**I. Polypharmacy**<br>(Definition: Large number of prescription medications.)	Prescription medications are defined as medications for which patient has a written prescription. They exclude alternative medications such as traditional Chinese medicine and other herbal supplements. Medication types with 2 separate dosages prescribed (e.g., warfarin 2 mg and 3 mg pills) should be counted as 1 medication.
