# Supplementary material for: Validation and application of a needs‐based segmentation tool for cross‐country comparisons
Source: Health Serv Res. 2021 Nov 10;56(Suppl 3):1394–404. doi: 10.1111/1475-6773.13873 (PMC8579203; doi:10.1111/1475-6773.13873)
Supplement: Supplementary file 2 — Data S2. Supporting information. [file HESR-56-1394-s001.docx]

Table 1: Wave 1 of CCSST-assigned Global Impressions (GI) segments with and without Complicating Factors (CFs) per country

|  | **Country** | | | | | | | | | | | |
| --- | --- | --- | --- | --- | --- | --- | --- | --- | --- | --- | --- | --- |
| **Characteristics** | **Austria** | **Belgium** | **Denmark** | **France** | **Germany** | **Greece** | **Israel** | **Italy** | **Netherlands** | **Spain** | **Sweden** | **Switzerland** |
| **N** | 1563 | 3810 | 1706 | 3122 | 2994 | 2897 | 2449 | 2552 | 2968 | 2316 | 3049 | 995 |
| **Gender** |  |  |  |  |  |  |  |  |  |  |  |  |
| Female (n, %) | 919 (58.8) | 2076 (54.49) | 934 (54.75) | 1766 (56.57) | 1622 (54.18) | 1655 (57.13) | 1375 (56.15) | 1423 (55.76) | 1605 (54.08) | 1348 (58.2) | 1638 (53.72) | 544 (54.67) |
| Male (n, %) | 644 (41.2) | 1734 (45.51) | 772 (45.25) | 1356 (43.43) | 1372 (45.82) | 1242 (42.87) | 1074 (43.85) | 1129 (44.24) | 1363 (45.92) | 968 (41.8) | 1411 (46.28) | 451 (45.33) |
| **Age** |  |  |  |  |  |  |  |  |  |  |  |  |
| 50-59 (n, %) | 475 (31.37) | 1447 (39.54) | 642 (40.25) | 1186 (40.04) | 1024 (35.13) | 1051 (39.74) | 947 (40.45) | 836 (33.57) | 1209 (42.53) | 712 (31.42) | 1081 (36.24) | 342 (36.62) |
| 60-69 (n, %) | 570 (37.65) | 1032 (28.2) | 457 (28.65) | 787 (26.57) | 1120 (38.42) | 777 (29.38) | 729 (31.14) | 948 (38.07) | 885 (31.13) | 672 (29.66) | 972 (32.58) | 284 (30.41) |
| 70-79 (n, %) | 328 (21.66) | 838 (22.9) | 328 (20.56) | 666 (22.48) | 582 (19.97) | 539 (20.38) | 470 (20.08) | 537 (21.57) | 529 (18.61) | 603 (26.61) | 631 (21.15) | 205 (21.95) |
| 80+ (n, %) | 141 (9.31) | 343 (9.37) | 168 (10.53) | 323 (10.9) | 189 (6.48) | 278 (10.51) | 195 (8.33) | 169 (6.79) | 220 (7.74) | 279 (12.31) | 299 (10.02) | 103 (11.03) |
| **Global Impression** |  |  |  |  |  |  |  |  |  |  |  |  |
| Healthy (n, %) | 471 (30.13) | 930 (24.41) | 525 (30.77) | 682 (21.84) | 927 (30.96) | 883 (30.48) | 562 (22.95) | 546 (21.39) | 1076 (36.25) | 470 (20.29) | 1051 (34.47) | 408 (41.01) |
| Chronic asymptomatic (n, %) | 334 (21.37) | 1146 (30.08) | 359 (21.04) | 892 (28.57) | 591 (19.74) | 933 (32.21) | 715 (29.2) | 759 (29.74) | 519 (17.49) | 533 (23.01) | 616 (20.2) | 245 (24.62) |
| Chronic symptomatic (n, %) | 497 (31.8) | 1095 (28.74) | 541 (31.71) | 839 (26.87) | 1005 (33.57) | 625 (21.57) | 590 (24.09) | 648 (25.39) | 902 (30.39) | 578 (24.96) | 994 (32.6) | 240 (24.12) |
| Long course of decline (n, %) | 114 (7.29) | 349 (9.16) | 140 (8.21) | 374 (11.98) | 177 (5.91) | 277 (9.56) | 345 (14.09) | 387 (15.16) | 193 (6.5) | 632 (27.29) | 142 (4.66) | 57 (5.73) |
| Limited reserve with serious exacerbation (n, %) | 97 (6.21) | 266 (6.98) | 122 (7.15) | 225 (7.21) | 219 (7.31) | 108 (3.73) | 181 (7.39) | 171 (6.7) | 214 (7.21) | 75 (3.24) | 203 (6.66) | 33 (3.32) |
| Unknown (n, %) | 50 (3.2) | 24 (0.63) | 19 (1.11) | 110 (3.52) | 75 (2.51) | 71 (2.45) | 56 (2.29) | 41 (1.61) | 64 (2.16) | 28 (1.21) | 43 (1.41) | 12 (1.21) |
| **Complicating Factors** |  |  |  |  |  |  |  |  |  |  |  |  |
| Not complicated (n, %) | 912 (58.35) | 1913 (50.21) | 888 (52.05) | 1552 (49.71) | 1626 (54.31) | 1532 (52.88) | 1178 (48.1) | 1457 (57.09) | 1574 (53.03) | 1210 (52.25) | 1712 (56.15) | 616 (61.91) |
| Complicated (n, %) | 647 (41.39) | 1895 (49.74) | 817 (47.89) | 1562 (50.03) | 1365 (45.59) | 1364 (47.08) | 1258 (51.37) | 1091 (42.75) | 1377 (46.39) | 1095 (47.28) | 1337 (43.85) | 378 (37.99) |
| Unknown (n, %) | 4 (0.26) | 2 (0.05) | 1 (0.06) | 8 (0.26) | 3 (0.1) | 1 (0.03) | 13 (0.53) | 4 (0.16) | 17 (0.57) | 11 (0.47) |  | 1 (0.1) |
| **Health State** |  |  |  |  |  |  |  |  |  |  |  |  |
| Healthy, not complicated (n, %) | 307 (19.64) | 522 (13.7) | 334 (19.58) | 411 (13.16) | 573 (19.14) | 524 (18.09) | 350 (14.29) | 360 (14.11) | 646 (21.77) | 336 (14.51) | 695 (22.79) | 272 (27.34) |
| Healthy, complicated (n, %) | 164 (10.49) | 408 (10.71) | 191 (11.2) | 271 (8.68) | 354 (11.82) | 359 (12.39) | 212 (8.66) | 186 (7.29) | 430 (14.49) | 134 (5.79) | 356 (11.68) | 136 (13.67) |
| Chronic asymptomatic, not complicated (n, %) | 243 (15.55) | 711 (18.66) | 228 (13.36) | 557 (17.84) | 383 (12.79) | 567 (19.57) | 448 (18.29) | 521 (20.42) | 331 (11.15) | 378 (16.32) | 406 (13.32) | 163 (16.38) |
| Chronic asymptomatic, complicated (n, %) | 91 (5.82) | 435 (11.42) | 131 (7.68) | 335 (10.73) | 208 (6.95) | 366 (12.63) | 267 (10.9) | 238 (9.33) | 188 (6.33) | 155 (6.69) | 210 (6.89) | 82 (8.24) |
| Chronic symptomatic, not complicated (n, %) | 286 (18.3) | 537 (14.09) | 269 (15.77) | 431 (13.81) | 546 (18.24) | 304 (10.49) | 267 (10.9) | 373 (14.62) | 487 (16.41) | 274 (11.83) | 521 (17.09) | 141 (14.17) |
| Chronic symptomatic, complicated (n, %) | 211 (13.5) | 558 (14.65) | 272 (15.94) | 408 (13.07) | 459 (15.33) | 321 (11.08) | 323 (13.19) | 275 (10.78) | 415 (13.98) | 304 (13.13) | 473 (15.51) | 99 (9.95) |
| Long course of decline, not complicated (n, %) | 31 (1.98) | 91 (2.39) | 33 (1.93) | 98 (3.14) | 47 (1.57) | 90 (3.11) | 85 (3.47) | 152 (5.96) | 48 (1.62) | 211 (9.11) | 31 (1.02) | 19 (1.91) |
| Long course of decline, complicated (n, %) | 83 (5.31) | 258 (6.77) | 107 (6.27) | 276 (8.84) | 130 (4.34) | 187 (6.45) | 260 (10.62) | 235 (9.21) | 145 (4.89) | 421 (18.18) | 111 (3.64) | 38 (3.82) |
| Limited reserve with serious exacerbation, not complicated (n, %) | 23 (1.47) | 44 (1.15) | 17 (1) | 38 (1.22) | 46 (1.54) | 15 (0.52) | 14 (0.57) | 30 (1.18) | 53 (1.79) | 8 (0.35) | 44 (1.44) | 15 (1.51) |
| Limited reserve with serious exacerbation, complicated (n, %) | 74 (4.73) | 222 (5.83) | 105 (6.15) | 187 (5.99) | 173 (5.78) | 93 (3.21) | 167 (6.82) | 141 (5.53) | 161 (5.42) | 67 (2.89) | 159 (5.21) | 18 (1.81) |
| Unknown (n, %) | 50 (3.2) | 24 (0.63) | 19 (1.11) | 110 (3.52) | 75 (2.51) | 71 (2.45) | 56 (2.29) | 41 (1.61) | 64 (2.16) | 28 (1.21) | 43 (1.41) | 12 (1.21) |

Table 2: Wave 2 of CCSST-assigned Global Impressions (GI) segments with and without Complicating Factors (CFs) per country

|  | **Country** | | | | | | | | | | | | | |
| --- | --- | --- | --- | --- | --- | --- | --- | --- | --- | --- | --- | --- | --- | --- |
| **Characteristics** | **Austria** | **Belgium** | **Czech Republic** | **Denmark** | **France** | **Germany** | **Greece** | **Israel** | **Italy** | **Netherlands** | **Poland** | **Spain** | **Sweden** | **Switzerland** |
| **N** | 1197 | 3227 | 2736 | 2630 | 2990 | 2628 | 3411 | 2447 | 2984 | 2683 | 2466 | 2423 | 2796 | 1498 |
| **Gender** |  |  |  |  |  |  |  |  |  |  |  |  |  |  |
| Female (n, %) | 713 (59.57) | 1765 (54.69) | 1588 (58.04) | 1447 (55.02) | 1711 (57.22) | 1424 (54.19) | 1948 (57.11) | 1388 (56.72) | 1639 (54.93) | 1464 (54.57) | 1391 (56.41) | 1343 (55.43) | 1519 (54.33) | 840 (56.07) |
| Male (n, %) | 484 (40.43) | 1462 (45.31) | 1148 (41.96) | 1183 (44.98) | 1279 (42.78) | 1204 (45.81) | 1463 (42.89) | 1059 (43.28) | 1345 (45.07) | 1219 (45.43) | 1075 (43.59) | 1080 (44.57) | 1277 (45.67) | 658 (43.93) |
| **Age** |  |  |  |  |  |  |  |  |  |  |  |  |  |  |
| 50-59 (n, %) | 308 (26.19) | 1177 (37.66) | 1021 (38.67) | 994 (39.21) | 1103 (38.41) | 884 (34.3) | 1215 (37.96) | 568 (23.53) | 903 (30.98) | 1029 (39.13) | 1010 (41.84) | 712 (30.05) | 791 (28.7) | 524 (36.09) |
| 60-69 (n, %) | 454 (38.61) | 926 (29.63) | 879 (33.3) | 783 (30.89) | 811 (28.24) | 942 (36.55) | 936 (29.24) | 847 (35.09) | 1070 (36.71) | 879 (33.42) | 697 (28.87) | 680 (28.7) | 1039 (37.7) | 477 (32.85) |
| 70-79 (n, %) | 286 (24.32) | 684 (21.89) | 514 (19.47) | 502 (19.8) | 640 (22.28) | 539 (20.92) | 735 (22.96) | 678 (28.09) | 726 (24.91) | 514 (19.54) | 516 (21.38) | 650 (27.44) | 579 (21.01) | 301 (20.73) |
| 80+ (n, %) | 128 (10.88) | 338 (10.82) | 226 (8.56) | 256 (10.1) | 318 (11.07) | 212 (8.23) | 315 (9.84) | 321 (13.3) | 216 (7.41) | 208 (7.91) | 191 (7.91) | 327 (13.8) | 347 (12.59) | 150 (10.33) |
| **Global Impression** |  |  |  |  |  |  |  |  |  |  |  |  |  |  |
| Healthy (n, %) | 250 (20.89) | 536 (16.61) | 742 (27.12) | 720 (27.38) | 513 (17.16) | 705 (26.83) | 971 (28.47) | 301 (12.3) | 478 (16.02) | 808 (30.12) | 404 (16.38) | 408 (16.84) | 776 (27.75) | 555 (37.05) |
| Chronic asymptomatic (n, %) | 280 (23.39) | 1108 (34.34) | 498 (18.2) | 735 (27.95) | 961 (32.14) | 580 (22.07) | 1198 (35.12) | 691 (28.24) | 972 (32.57) | 548 (20.42) | 479 (19.42) | 707 (29.18) | 675 (24.14) | 425 (28.37) |
| Chronic symptomatic (n, %) | 439 (36.68) | 1012 (31.36) | 973 (35.56) | 814 (30.95) | 841 (28.13) | 938 (35.69) | 730 (21.4) | 675 (27.58) | 797 (26.71) | 901 (33.58) | 789 (32) | 625 (25.79) | 975 (34.87) | 376 (25.1) |
| Long course of decline (n, %) | 109 (9.11) | 253 (7.84) | 220 (8.04) | 175 (6.65) | 349 (11.67) | 118 (4.49) | 326 (9.56) | 409 (16.71) | 440 (14.75) | 167 (6.22) | 416 (16.87) | 574 (23.69) | 134 (4.79) | 64 (4.27) |
| Limited reserve with serious exacerbation (n, %) | 102 (8.52) | 306 (9.48) | 271 (9.9) | 174 (6.62) | 235 (7.86) | 238 (9.06) | 139 (4.08) | 315 (12.87) | 248 (8.31) | 241 (8.98) | 355 (14.4) | 81 (3.34) | 199 (7.12) | 58 (3.87) |
| Unknown (n, %) | 17 (1.42) | 12 (0.37) | 32 (1.17) | 12 (0.46) | 91 (3.04) | 49 (1.86) | 47 (1.38) | 56 (2.29) | 49 (1.64) | 18 (0.67) | 23 (0.93) | 28 (1.16) | 37 (1.32) | 20 (1.34) |
| **Complicating Factors** |  |  |  |  |  |  |  |  |  |  |  |  |  |  |
| Not complicated (n, %) | 640 (53.47) | 1603 (49.67) | 1440 (52.63) | 1411 (53.65) | 1573 (52.61) | 1422 (54.11) | 1704 (49.96) | 872 (35.64) | 1475 (49.43) | 1522 (56.73) | 1075 (43.59) | 1156 (47.71) | 1594 (57.01) | 939 (62.68) |
| Complicated (n, %) | 555 (46.37) | 1623 (50.29) | 1292 (47.22) | 1218 (46.31) | 1400 (46.82) | 1200 (45.66) | 1703 (49.93) | 1563 (63.87) | 1505 (50.44) | 1160 (43.24) | 1382 (56.04) | 1265 (52.21) | 1198 (42.85) | 559 (37.32) |
| Unknown (n, %) | 2 (0.17) | 1 (0.03) | 4 (0.15) | 1 (0.04) | 17 (0.57) | 6 (0.23) | 4 (0.12) | 12 (0.49) | 4 (0.13) | 1 (0.04) | 9 (0.36) | 2 (0.08) | 4 (0.14) | 0 (0) |
| **Health State** |  |  |  |  |  |  |  |  |  |  |  |  |  |  |
| Healthy, not complicated (n, %) | 151 (12.61) | 331 (10.26) | 457 (16.7) | 424 (16.12) | 337 (11.27) | 413 (15.72) | 545 (15.98) | 157 (6.42) | 264 (8.85) | 522 (19.46) | 253 (10.26) | 227 (9.37) | 515 (18.42) | 389 (25.97) |
| Healthy, complicated (n, %) | 99 (8.27) | 205 (6.35) | 285 (10.42) | 296 (11.25) | 176 (5.89) | 292 (11.11) | 426 (12.49) | 144 (5.88) | 214 (7.17) | 286 (10.66) | 151 (6.12) | 181 (7.47) | 261 (9.33) | 166 (11.08) |
| Chronic asymptomatic, not complicated (n, %) | 196 (16.37) | 690 (21.38) | 331 (12.1) | 487 (18.52) | 625 (20.9) | 376 (14.31) | 699 (20.49) | 371 (15.16) | 584 (19.57) | 362 (13.49) | 319 (12.94) | 455 (18.78) | 456 (16.31) | 290 (19.36) |
| Chronic asymptomatic, complicated (n, %) | 84 (7.02) | 418 (12.95) | 167 (6.1) | 248 (9.43) | 336 (11.24) | 204 (7.76) | 499 (14.63) | 320 (13.08) | 388 (13) | 186 (6.93) | 160 (6.49) | 252 (10.4) | 219 (7.83) | 135 (9.01) |
| Chronic symptomatic, not complicated (n, %) | 248 (20.72) | 492 (15.25) | 512 (18.71) | 422 (16.05) | 460 (15.38) | 532 (20.24) | 337 (9.88) | 243 (9.93) | 424 (14.21) | 520 (19.38) | 348 (14.11) | 286 (11.8) | 530 (18.96) | 214 (14.29) |
| Chronic symptomatic, complicated (n, %) | 191 (15.96) | 520 (16.11) | 461 (16.85) | 392 (14.9) | 381 (12.74) | 406 (15.45) | 393 (11.52) | 432 (17.65) | 373 (12.5) | 381 (14.2) | 441 (17.88) | 339 (13.99) | 445 (15.92) | 162 (10.81) |
| Long course of decline, not complicated (n, %) | 22 (1.84) | 47 (1.46) | 75 (2.74) | 43 (1.63) | 98 (3.28) | 32 (1.22) | 82 (2.4) | 72 (2.94) | 152 (5.09) | 40 (1.49) | 100 (4.06) | 170 (7.02) | 31 (1.11) | 19 (1.27) |
| Long course of decline, complicated (n, %) | 87 (7.27) | 206 (6.38) | 145 (5.3) | 132 (5.02) | 251 (8.39) | 86 (3.27) | 244 (7.15) | 337 (13.77) | 288 (9.65) | 127 (4.73) | 316 (12.81) | 404 (16.67) | 103 (3.68) | 45 (3) |
| Limited reserve with serious exacerbation, not complicated (n, %) | 14 (1.17) | 37 (1.15) | 57 (2.08) | 31 (1.18) | 30 (1) | 51 (1.94) | 17 (0.5) | 20 (0.82) | 36 (1.21) | 72 (2.68) | 49 (1.99) | 10 (0.41) | 40 (1.43) | 22 (1.47) |
| Limited reserve with serious exacerbation, complicated (n, %) | 88 (7.35) | 269 (8.34) | 214 (7.82) | 143 (5.44) | 205 (6.86) | 187 (7.12) | 122 (3.58) | 295 (12.06) | 212 (7.1) | 169 (6.3) | 306 (12.41) | 71 (2.93) | 159 (5.69) | 36 (2.4) |
| Unknown (n, %) | 17 (1.42) | 12 (0.37) | 32 (1.17) | 12 (0.46) | 91 (3.04) | 49 (1.86) | 47 (1.38) | 56 (2.29) | 49 (1.64) | 18 (0.67) | 23 (0.93) | 28 (1.16) | 37 (1.32) | 20 (1.34) |

Table 3: Wave 4 of CCSST-assigned Global Impressions (GI) segments with and without Complicating Factors (CFs) per country

|  | **Country** | | | | | | | | | | | |
| --- | --- | --- | --- | --- | --- | --- | --- | --- | --- | --- | --- | --- |
| **Characteristics** | **Austria** | **Belgium** | **Czech Republic** | **Denmark** | **France** | **Germany** | **Italy** | **Netherlands** | **Poland** | **Spain** | **Sweden** | **Switzerland** |
| **N** | 5247 | 5322 | 5521 | 2287 | 5851 | 1619 | 3570 | 2789 | 1733 | 3727 | 1969 | 3786 |
| **Gender** |  |  |  |  |  |  |  |  |  |  |  |  |
| Female (n, %) | 3041 (57.96) | 2951 (55.45) | 3232 (58.54) | 1246 (54.48) | 3354 (57.32) | 863 (53.3) | 1970 (55.18) | 1559 (55.9) | 978 (56.43) | 2060 (55.27) | 1069 (54.29) | 2091 (55.23) |
| Male (n, %) | 2206 (42.04) | 2371 (44.55) | 2289 (41.46) | 1041 (45.52) | 2497 (42.68) | 756 (46.7) | 1600 (44.82) | 1230 (44.1) | 755 (43.57) | 1667 (44.73) | 900 (45.71) | 1695 (44.77) |
| **Age** |  |  |  |  |  |  |  |  |  |  |  |  |
| 50-59 (n, %) | 1552 (30.5) | 1955 (37.78) | 1611 (29.99) | 849 (38.14) | 1898 (33.68) | 327 (20.34) | 903 (25.76) | 801 (29.15) | 427 (24.81) | 1015 (27.82) | 258 (13.14) | 1278 (34.84) |
| 60-69 (n, %) | 1768 (34.75) | 1560 (30.14) | 2118 (39.43) | 718 (32.26) | 1733 (30.75) | 609 (37.87) | 1278 (36.46) | 1091 (39.7) | 700 (40.67) | 1055 (28.92) | 816 (41.57) | 1218 (33.21) |
| 70-79 (n, %) | 1252 (24.61) | 1023 (19.77) | 1174 (21.85) | 402 (18.06) | 1230 (21.83) | 489 (30.41) | 970 (27.67) | 577 (21) | 390 (22.66) | 992 (27.19) | 580 (29.55) | 795 (21.67) |
| 80+ (n, %) | 516 (10.14) | 637 (12.31) | 469 (8.73) | 257 (11.55) | 774 (13.74) | 183 (11.38) | 354 (10.1) | 279 (10.15) | 204 (11.85) | 586 (16.06) | 309 (15.74) | 377 (10.28) |
| **Global Impression** |  |  |  |  |  |  |  |  |  |  |  |  |
| Healthy (n, %) | 1353 (25.79) | 881 (16.55) | 1055 (19.11) | 518 (22.65) | 1010 (17.26) | 240 (14.82) | 597 (16.72) | 720 (25.82) | 189 (10.91) | 589 (15.8) | 403 (20.47) | 1270 (33.54) |
| Chronic asymptomatic (n, %) | 1169 (22.28) | 1384 (26.01) | 1070 (19.38) | 679 (29.69) | 1700 (29.05) | 344 (21.25) | 1089 (30.5) | 542 (19.43) | 399 (23.02) | 1095 (29.38) | 516 (26.21) | 1101 (29.08) |
| Chronic symptomatic (n, %) | 1755 (33.45) | 1953 (36.7) | 2338 (42.35) | 794 (34.72) | 1894 (32.37) | 740 (45.71) | 959 (26.86) | 1039 (37.25) | 593 (34.22) | 873 (23.42) | 721 (36.62) | 1061 (28.02) |
| Long course of decline (n, %) | 447 (8.52) | 520 (9.77) | 372 (6.74) | 137 (5.99) | 627 (10.72) | 92 (5.68) | 551 (15.43) | 184 (6.6) | 297 (17.14) | 963 (25.84) | 155 (7.87) | 175 (4.62) |
| Limited reserve with serious exacerbation (n, %) | 366 (6.98) | 521 (9.79) | 594 (10.76) | 139 (6.08) | 480 (8.2) | 191 (11.8) | 318 (8.91) | 281 (10.08) | 244 (14.08) | 170 (4.56) | 155 (7.87) | 135 (3.57) |
| Unknown (n, %) | 157 (2.99) | 63 (1.18) | 92 (1.67) | 20 (0.87) | 140 (2.39) | 12 (0.74) | 56 (1.57) | 23 (0.82) | 11 (0.63) | 37 (0.99) | 19 (0.96) | 44 (1.16) |
| **Complicating Factors** |  |  |  |  |  |  |  |  |  |  |  |  |
| Not complicated (n, %) | 2771 (52.81) | 2598 (48.82) | 2728 (49.41) | 1254 (54.83) | 3043 (52.01) | 808 (49.91) | 1782 (49.92) | 1507 (54.03) | 756 (43.62) | 1668 (44.75) | 1067 (54.19) | 2219 (58.61) |
| Complicated (n, %) | 2463 (46.94) | 2711 (50.94) | 2774 (50.24) | 1028 (44.95) | 2772 (47.38) | 811 (50.09) | 1780 (49.86) | 1279 (45.86) | 974 (56.2) | 2050 (55) | 900 (45.71) | 1565 (41.34) |
| Unknown (n, %) | 13 (0.25) | 13 (0.24) | 19 (0.34) | 5 (0.22) | 36 (0.62) | 0 (0) | 8 (0.22) | 3 (0.11) | 3 (0.17) | 9 (0.24) | 2 (0.1) | 2 (0.05) |
| **Health State** |  |  |  |  |  |  |  |  |  |  |  |  |
| Healthy, not complicated (n, %) | 900 (17.15) | 594 (11.16) | 640 (11.59) | 321 (14.04) | 666 (11.38) | 147 (9.08) | 361 (10.11) | 444 (15.92) | 91 (5.25) | 323 (8.67) | 256 (13) | 803 (21.21) |
| Healthy, complicated (n, %) | 453 (8.63) | 287 (5.39) | 415 (7.52) | 197 (8.61) | 344 (5.88) | 93 (5.74) | 236 (6.61) | 276 (9.9) | 98 (5.65) | 266 (7.14) | 147 (7.47) | 467 (12.33) |
| Chronic asymptomatic, not complicated (n, %) | 784 (14.94) | 877 (16.48) | 686 (12.43) | 448 (19.59) | 1096 (18.73) | 220 (13.59) | 641 (17.96) | 351 (12.59) | 245 (14.14) | 660 (17.71) | 344 (17.47) | 736 (19.44) |
| Chronic asymptomatic, complicated (n, %) | 385 (7.34) | 507 (9.53) | 384 (6.96) | 231 (10.1) | 604 (10.32) | 124 (7.66) | 448 (12.55) | 191 (6.85) | 154 (8.89) | 435 (11.67) | 172 (8.74) | 365 (9.64) |
| Chronic symptomatic, not complicated (n, %) | 922 (17.57) | 903 (16.97) | 1172 (21.23) | 424 (18.54) | 969 (16.56) | 386 (23.84) | 498 (13.95) | 571 (20.47) | 284 (16.39) | 376 (10.09) | 378 (19.2) | 579 (15.29) |
| Chronic symptomatic, complicated (n, %) | 833 (15.88) | 1050 (19.73) | 1166 (21.12) | 370 (16.18) | 925 (15.81) | 354 (21.87) | 461 (12.91) | 468 (16.78) | 309 (17.83) | 497 (13.34) | 343 (17.42) | 482 (12.73) |
| Long course of decline, not complicated (n, %) | 75 (1.43) | 120 (2.25) | 86 (1.56) | 31 (1.36) | 201 (3.44) | 23 (1.42) | 201 (5.63) | 51 (1.83) | 95 (5.48) | 284 (7.62) | 50 (2.54) | 54 (1.43) |
| Long course of decline, complicated (n, %) | 372 (7.09) | 400 (7.52) | 286 (5.18) | 106 (4.63) | 426 (7.28) | 69 (4.26) | 350 (9.8) | 133 (4.77) | 202 (11.66) | 679 (18.22) | 105 (5.33) | 121 (3.2) |
| Limited reserve with serious exacerbation, not complicated (n, %) | 56 (1.07) | 88 (1.65) | 122 (2.21) | 25 (1.09) | 82 (1.4) | 29 (1.79) | 58 (1.62) | 83 (2.98) | 39 (2.25) | 18 (0.48) | 33 (1.68) | 30 (0.79) |
| Limited reserve with serious exacerbation, complicated (n, %) | 310 (5.91) | 433 (8.14) | 472 (8.55) | 114 (4.98) | 398 (6.8) | 162 (10.01) | 260 (7.28) | 198 (7.1) | 205 (11.83) | 152 (4.08) | 122 (6.2) | 105 (2.77) |
| Unknown (n, %) | 157 (2.99) | 63 (1.18) | 92 (1.67) | 20 (0.87) | 140 (2.39) | 12 (0.74) | 56 (1.57) | 23 (0.82) | 11 (0.63) | 37 (0.99) | 19 (0.96) | 44 (1.16) |

Table 4: Wave 5 of CCSST-assigned Global Impressions (GI) segments with and without Complicating Factors (CFs) per country

|  | **Country** | | | | | | | | | | | |
| --- | --- | --- | --- | --- | --- | --- | --- | --- | --- | --- | --- | --- |
| **Characteristics** | **Austria** | **Belgium** | **Czech Republic** | **Denmark** | **France** | **Germany** | **Israel** | **Italy** | **Netherlands** | **Spain** | **Sweden** | **Switzerland** |
| **N** | 4378 | 5637 | 5640 | 4146 | 4506 | 5751 | 2599 | 4744 | 4167 | 6693 | 4555 | 3049 |
| **Gender** |  |  |  |  |  |  |  |  |  |  |  |  |
| Female (n, %) | 2516 (57.47) | 3125 (55.44) | 3324 (58.94) | 2243 (54.1) | 2586 (57.39) | 3061 (53.23) | 1461 (56.21) | 2613 (55.08) | 2303 (55.27) | 3637 (54.34) | 2438 (53.52) | 1682 (55.17) |
| Male (n, %) | 1862 (42.53) | 2512 (44.56) | 2316 (41.06) | 1903 (45.9) | 1920 (42.61) | 2690 (46.77) | 1138 (43.79) | 2131 (44.92) | 1864 (44.73) | 3056 (45.66) | 2117 (46.48) | 1367 (44.83) |
| **Age** |  |  |  |  |  |  |  |  |  |  |  |  |
| 50-59 (n, %) | 1073 (25.03) | 1903 (34.42) | 1340 (24.21) | 1436 (35.4) | 1190 (26.9) | 2015 (36.09) | 631 (24.64) | 1257 (27) | 1178 (28.61) | 1714 (26.11) | 827 (18.32) | 878 (29.39) |
| 60-69 (n, %) | 1504 (35.08) | 1780 (32.19) | 2263 (40.88) | 1389 (34.25) | 1552 (35.08) | 1787 (32.01) | 921 (35.96) | 1589 (34.14) | 1616 (39.24) | 2032 (30.96) | 1839 (40.74) | 1056 (35.35) |
| 70-79 (n, %) | 1201 (28.01) | 1119 (20.24) | 1390 (25.11) | 798 (19.67) | 981 (22.17) | 1320 (23.64) | 642 (25.07) | 1276 (27.41) | 900 (21.86) | 1618 (24.65) | 1259 (27.89) | 722 (24.17) |
| 80+ (n, %) | 509 (11.87) | 727 (13.15) | 543 (9.81) | 433 (10.68) | 701 (15.85) | 461 (8.26) | 367 (14.33) | 533 (11.45) | 424 (10.3) | 1200 (18.28) | 589 (13.05) | 331 (11.08) |
| **Global Impression** |  |  |  |  |  |  |  |  |  |  |  |  |
| Healthy (n, %) | 750 (17.13) | 792 (14.05) | 778 (13.79) | 969 (23.37) | 548 (12.16) | 1213 (21.09) | 386 (14.85) | 757 (15.96) | 1053 (25.27) | 1142 (17.06) | 1038 (22.79) | 782 (25.65) |
| Chronic asymptomatic (n, %) | 1182 (27) | 1712 (30.37) | 1532 (27.16) | 1234 (29.76) | 1439 (31.94) | 1394 (24.24) | 777 (29.9) | 1464 (30.86) | 882 (21.17) | 1982 (29.61) | 1276 (28.01) | 1049 (34.4) |
| Chronic symptomatic (n, %) | 1568 (35.82) | 1993 (35.36) | 2203 (39.06) | 1442 (34.78) | 1529 (33.93) | 2216 (38.53) | 666 (25.63) | 1259 (26.54) | 1518 (36.43) | 1554 (23.22) | 1667 (36.6) | 907 (29.75) |
| Long course of decline (n, %) | 377 (8.61) | 602 (10.68) | 506 (8.97) | 246 (5.93) | 518 (11.5) | 333 (5.79) | 358 (13.77) | 772 (16.27) | 263 (6.31) | 1643 (24.55) | 245 (5.38) | 169 (5.54) |
| Limited reserve with serious exacerbation (n, %) | 377 (8.61) | 505 (8.96) | 568 (10.07) | 233 (5.62) | 447 (9.92) | 536 (9.32) | 308 (11.85) | 427 (9) | 429 (10.3) | 289 (4.32) | 301 (6.61) | 114 (3.74) |
| Unknown (n, %) | 124 (2.83) | 33 (0.59) | 53 (0.94) | 22 (0.53) | 25 (0.55) | 59 (1.03) | 104 (4) | 65 (1.37) | 22 (0.53) | 83 (1.24) | 28 (0.61) | 28 (0.92) |
| **Complicating Factors** |  |  |  |  |  |  |  |  |  |  |  |  |
| Not complicated (n, %) | 2272 (51.9) | 2821 (50.04) | 2753 (48.81) | 2276 (54.9) | 2299 (51.02) | 3045 (52.95) | 1091 (41.98) | 2280 (48.06) | 2325 (55.8) | 2995 (44.75) | 2572 (56.47) | 1825 (59.86) |
| Complicated (n, %) | 2097 (47.9) | 2808 (49.81) | 2877 (51.01) | 1866 (45.01) | 2201 (48.85) | 2694 (46.84) | 1495 (57.52) | 2461 (51.88) | 1838 (44.11) | 3684 (55.04) | 1976 (43.38) | 1223 (40.11) |
| Unknown (n, %) | 9 (0.21) | 8 (0.14) | 10 (0.18) | 4 (0.1) | 6 (0.13) | 12 (0.21) | 13 (0.5) | 3 (0.06) | 4 (0.1) | 14 (0.21) | 7 (0.15) | 1 (0.03) |
| **Health State** |  |  |  |  |  |  |  |  |  |  |  |  |
| Healthy, not complicated (n, %) | 474 (10.83) | 517 (9.17) | 469 (8.32) | 611 (14.74) | 338 (7.5) | 792 (13.77) | 241 (9.27) | 426 (8.98) | 672 (16.13) | 651 (9.73) | 695 (15.26) | 527 (17.28) |
| Healthy, complicated (n, %) | 276 (6.3) | 275 (4.88) | 309 (5.48) | 358 (8.63) | 210 (4.66) | 421 (7.32) | 145 (5.58) | 331 (6.98) | 381 (9.14) | 491 (7.34) | 343 (7.53) | 255 (8.36) |
| Chronic asymptomatic, not complicated (n, %) | 775 (17.7) | 1114 (19.76) | 937 (16.61) | 791 (19.08) | 938 (20.82) | 888 (15.44) | 436 (16.78) | 887 (18.7) | 563 (13.51) | 1158 (17.3) | 827 (18.16) | 709 (23.25) |
| Chronic asymptomatic, complicated (n, %) | 407 (9.3) | 598 (10.61) | 595 (10.55) | 443 (10.68) | 501 (11.12) | 506 (8.8) | 341 (13.12) | 577 (12.16) | 319 (7.66) | 824 (12.31) | 449 (9.86) | 340 (11.15) |
| Chronic symptomatic, not complicated (n, %) | 844 (19.28) | 980 (17.39) | 1119 (19.84) | 771 (18.6) | 798 (17.71) | 1142 (19.86) | 277 (10.66) | 642 (13.53) | 902 (21.65) | 734 (10.97) | 907 (19.91) | 490 (16.07) |
| Chronic symptomatic, complicated (n, %) | 724 (16.54) | 1013 (17.97) | 1084 (19.22) | 671 (16.18) | 731 (16.22) | 1074 (18.68) | 389 (14.97) | 617 (13.01) | 616 (14.78) | 820 (12.25) | 760 (16.68) | 417 (13.68) |
| Long course of decline, not complicated (n, %) | 78 (1.78) | 137 (2.43) | 131 (2.32) | 62 (1.5) | 161 (3.57) | 89 (1.55) | 71 (2.73) | 232 (4.89) | 59 (1.42) | 431 (6.44) | 87 (1.91) | 54 (1.77) |
| Long course of decline, complicated (n, %) | 299 (6.83) | 465 (8.25) | 375 (6.65) | 184 (4.44) | 357 (7.92) | 244 (4.24) | 287 (11.04) | 540 (11.38) | 204 (4.9) | 1212 (18.11) | 158 (3.47) | 115 (3.77) |
| Limited reserve with serious exacerbation, not complicated (n, %) | 54 (1.23) | 62 (1.1) | 81 (1.44) | 36 (0.87) | 58 (1.29) | 117 (2.03) | 23 (0.88) | 71 (1.5) | 122 (2.93) | 11 (0.16) | 49 (1.08) | 29 (0.95) |
| Limited reserve with serious exacerbation, complicated (n, %) | 323 (7.38) | 443 (7.86) | 487 (8.63) | 197 (4.75) | 389 (8.63) | 419 (7.29) | 285 (10.97) | 356 (7.5) | 307 (7.37) | 278 (4.15) | 252 (5.53) | 85 (2.79) |
| Unknown (n, %) | 124 (2.83) | 33 (0.59) | 53 (0.94) | 22 (0.53) | 25 (0.55) | 59 (1.03) | 104 (4) | 65 (1.37) | 22 (0.53) | 83 (1.24) | 28 (0.61) | 28 (0.92) |

Table 5: Wave 6 of CCSST-assigned Global Impressions (GI) segments with and without Complicating Factors (CFs) per country

|  | **Country** | | | | | | | | | | | | |
| --- | --- | --- | --- | --- | --- | --- | --- | --- | --- | --- | --- | --- | --- |
| **Characteristics** | **Austria** | **Belgium** | **Czech Republic** | **Denmark** | **France** | **Germany** | **Greece** | **Israel** | **Italy** | **Poland** | **Spain** | **Sweden** | **Switzerland** |
| **N** | 3397 | 5815 | 4856 | 3733 | 3947 | 4412 | 4926 | 2035 | 5308 | 1826 | 5623 | 3906 | 2803 |
| **Gender** |  |  |  |  |  |  |  |  |  |  |  |  |  |
| Female (n, %) | 1976 (58.17) | 3242 (55.75) | 2905 (59.82) | 2016 (54) | 2267 (57.44) | 2335 (52.92) | 2806 (56.96) | 1176 (57.79) | 2917 (54.95) | 1034 (56.63) | 3115 (55.4) | 2119 (54.25) | 1545 (55.12) |
| Male (n, %) | 1421 (41.83) | 2573 (44.25) | 1951 (40.18) | 1717 (46) | 1680 (42.56) | 2077 (47.08) | 2120 (43.04) | 859 (42.21) | 2391 (45.05) | 792 (43.37) | 2508 (44.6) | 1787 (45.75) | 1258 (44.88) |
| **Age** |  |  |  |  |  |  |  |  |  |  |  |  |  |
| 50-59 (n, %) | 608 (18.11) | 1824 (32) | 901 (18.8) | 1230 (33.6) | 1009 (26.07) | 1276 (29.35) | 1262 (26.22) | 283 (14.06) | 1394 (26.75) | 495 (27.47) | 1072 (19.28) | 484 (12.47) | 608 (21.93) |
| 60-69 (n, %) | 1209 (36) | 1899 (33.32) | 1941 (40.5) | 1262 (34.47) | 1330 (34.37) | 1510 (34.74) | 1704 (35.4) | 819 (40.69) | 1793 (34.41) | 727 (40.34) | 1827 (32.86) | 1500 (38.65) | 1023 (36.9) |
| 70-79 (n, %) | 1077 (32.07) | 1178 (20.67) | 1396 (29.13) | 781 (21.33) | 870 (22.48) | 1140 (26.22) | 1183 (24.57) | 528 (26.23) | 1407 (27) | 369 (20.48) | 1472 (26.47) | 1286 (33.14) | 731 (26.37) |
| 80+ (n, %) | 464 (13.82) | 799 (14.02) | 555 (11.58) | 388 (10.6) | 661 (17.08) | 421 (9.68) | 665 (13.81) | 383 (19.03) | 617 (11.84) | 211 (11.71) | 1189 (21.38) | 611 (15.74) | 410 (14.79) |
| **Global Impression** |  |  |  |  |  |  |  |  |  |  |  |  |  |
| Healthy (n, %) | 423 (12.45) | 715 (12.3) | 480 (9.88) | 760 (20.36) | 418 (10.59) | 656 (14.87) | 1051 (21.34) | 267 (13.12) | 857 (16.15) | 214 (11.72) | 587 (10.44) | 692 (17.72) | 559 (19.94) |
| Chronic asymptomatic (n, %) | 922 (27.14) | 1714 (29.48) | 1281 (26.38) | 1101 (29.49) | 1239 (31.39) | 1058 (23.98) | 1796 (36.46) | 578 (28.4) | 1727 (32.54) | 419 (22.95) | 1871 (33.27) | 1103 (28.24) | 959 (34.21) |
| Chronic symptomatic (n, %) | 1363 (40.12) | 2191 (37.68) | 2139 (44.05) | 1450 (38.84) | 1462 (37.04) | 2009 (45.53) | 1126 (22.86) | 585 (28.75) | 1402 (26.41) | 678 (37.13) | 1588 (28.24) | 1521 (38.94) | 998 (35.6) |
| Long course of decline (n, %) | 254 (7.48) | 624 (10.73) | 328 (6.75) | 208 (5.57) | 403 (10.21) | 199 (4.51) | 659 (13.38) | 291 (14.3) | 768 (14.47) | 231 (12.65) | 1253 (22.28) | 255 (6.53) | 143 (5.1) |
| Limited reserve with serious exacerbation (n, %) | 360 (10.6) | 535 (9.2) | 605 (12.46) | 200 (5.36) | 392 (9.93) | 472 (10.7) | 226 (4.59) | 278 (13.66) | 458 (8.63) | 263 (14.4) | 255 (4.53) | 310 (7.94) | 136 (4.85) |
| Unknown (n, %) | 75 (2.21) | 36 (0.62) | 23 (0.47) | 14 (0.38) | 33 (0.84) | 18 (0.41) | 68 (1.38) | 36 (1.77) | 96 (1.81) | 21 (1.15) | 69 (1.23) | 25 (0.64) | 8 (0.29) |
| **Complicating Factors** |  |  |  |  |  |  |  |  |  |  |  |  |  |
| Not complicated (n, %) | 1793 (52.78) | 2933 (50.44) | 2597 (53.48) | 2290 (61.34) | 2039 (51.66) | 2530 (57.34) | 2577 (52.31) | 931 (45.75) | 2732 (51.47) | 845 (46.28) | 2580 (45.88) | 2264 (57.96) | 1711 (61.04) |
| Complicated (n, %) | 1599 (47.07) | 2874 (49.42) | 2256 (46.46) | 1441 (38.6) | 1901 (48.16) | 1878 (42.57) | 2348 (47.67) | 1098 (53.96) | 2565 (48.32) | 975 (53.4) | 3034 (53.96) | 1635 (41.86) | 1091 (38.92) |
| Unknown (n, %) | 5 (0.15) | 8 (0.14) | 3 (0.06) | 2 (0.05) | 7 (0.18) | 4 (0.09) | 1 (0.02) | 6 (0.29) | 11 (0.21) | 6 (0.33) | 9 (0.16) | 7 (0.18) | 1 (0.04) |
| **Health State** |  |  |  |  |  |  |  |  |  |  |  |  |  |
| Healthy, not complicated (n, %) | 290 (8.54) | 495 (8.51) | 331 (6.82) | 522 (13.98) | 298 (7.55) | 450 (10.2) | 656 (13.32) | 167 (8.21) | 548 (10.32) | 134 (7.34) | 350 (6.22) | 465 (11.9) | 391 (13.95) |
| Healthy, complicated (n, %) | 133 (3.92) | 220 (3.78) | 149 (3.07) | 238 (6.38) | 120 (3.04) | 206 (4.67) | 395 (8.02) | 100 (4.91) | 309 (5.82) | 80 (4.38) | 237 (4.21) | 227 (5.81) | 168 (5.99) |
| Chronic asymptomatic, not complicated (n, %) | 611 (17.99) | 1145 (19.69) | 918 (18.9) | 785 (21.03) | 815 (20.65) | 715 (16.21) | 1122 (22.78) | 373 (18.33) | 1048 (19.74) | 254 (13.91) | 1143 (20.33) | 778 (19.92) | 659 (23.51) |
| Chronic asymptomatic, complicated (n, %) | 311 (9.16) | 569 (9.79) | 363 (7.48) | 316 (8.47) | 424 (10.74) | 343 (7.77) | 674 (13.68) | 205 (10.07) | 679 (12.79) | 165 (9.04) | 728 (12.95) | 325 (8.32) | 300 (10.7) |
| Chronic symptomatic, not complicated (n, %) | 768 (22.61) | 1091 (18.76) | 1154 (23.76) | 881 (23.6) | 770 (19.51) | 1198 (27.15) | 549 (11.14) | 281 (13.81) | 751 (14.15) | 335 (18.35) | 728 (12.95) | 870 (22.27) | 585 (20.87) |
| Chronic symptomatic, complicated (n, %) | 595 (17.52) | 1100 (18.92) | 985 (20.28) | 569 (15.24) | 692 (17.53) | 811 (18.38) | 577 (11.71) | 304 (14.94) | 651 (12.26) | 343 (18.78) | 860 (15.29) | 651 (16.67) | 413 (14.73) |
| Long course of decline, not complicated (n, %) | 61 (1.8) | 137 (2.36) | 85 (1.75) | 67 (1.79) | 105 (2.66) | 62 (1.41) | 200 (4.06) | 73 (3.59) | 302 (5.69) | 72 (3.94) | 325 (5.78) | 80 (2.05) | 45 (1.61) |
| Long course of decline, complicated (n, %) | 193 (5.68) | 487 (8.37) | 243 (5) | 141 (3.78) | 298 (7.55) | 137 (3.11) | 459 (9.32) | 218 (10.71) | 466 (8.78) | 159 (8.71) | 928 (16.5) | 175 (4.48) | 98 (3.5) |
| Limited reserve with serious exacerbation, not complicated (n, %) | 44 (1.3) | 55 (0.95) | 103 (2.12) | 30 (0.8) | 48 (1.22) | 100 (2.27) | 17 (0.35) | 25 (1.23) | 61 (1.15) | 45 (2.46) | 14 (0.25) | 61 (1.56) | 30 (1.07) |
| Limited reserve with serious exacerbation, complicated (n, %) | 316 (9.3) | 480 (8.25) | 502 (10.34) | 170 (4.55) | 344 (8.72) | 372 (8.43) | 209 (4.24) | 253 (12.43) | 397 (7.48) | 218 (11.94) | 241 (4.29) | 249 (6.37) | 106 (3.78) |
| Unknown (n, %) | 75 (2.21) | 36 (0.62) | 23 (0.47) | 14 (0.38) | 33 (0.84) | 18 (0.41) | 68 (1.38) | 36 (1.77) | 96 (1.81) | 21 (1.15) | 69 (1.23) | 25 (0.64) | 8 (0.29) |

Table 6: Wave 7 of CCSST-assigned Global Impressions (GI) segments with and without Complicating Factors (CFs) per country

|  | **Country** | | | | | | | | | | | |
| --- | --- | --- | --- | --- | --- | --- | --- | --- | --- | --- | --- | --- |
| **Characteristics** | **Austria** | **Belgium** | **Czech Republic** | **Denmark** | **France** | **Germany** | **Greece** | **Italy** | **Poland** | **Spain** | **Sweden** | **Switzerland** |
| **N** | 483 | 1567 | 921 | 1284 | 1143 | 836 | 1911 | 1570 | 1144 | 1280 | 1066 | 754 |
| **Gender** |  |  |  |  |  |  |  |  |  |  |  |  |
| Female (n, %) | 304 (62.94) | 888 (56.67) | 557 (60.48) | 726 (56.54) | 666 (58.27) | 453 (54.19) | 1105 (57.82) | 890 (56.69) | 663 (57.95) | 744 (58.13) | 613 (57.5) | 434 (57.56) |
| Male (n, %) | 179 (37.06) | 679 (43.33) | 364 (39.52) | 558 (43.46) | 477 (41.73) | 383 (45.81) | 806 (42.18) | 680 (43.31) | 481 (42.05) | 536 (41.88) | 453 (42.5) | 320 (42.44) |
| **Age** |  |  |  |  |  |  |  |  |  |  |  |  |
| 50-59 (n, %) | 7 (1.46) | 47 (3) | 23 (2.5) | 49 (3.82) | 31 (2.72) | 15 (1.79) | 124 (6.5) | 38 (2.42) | 33 (2.89) | 33 (2.58) | 11 (1.03) | 16 (2.14) |
| 60-69 (n, %) | 139 (28.9) | 631 (40.32) | 359 (39.02) | 596 (46.42) | 505 (44.3) | 369 (44.14) | 797 (41.79) | 559 (35.61) | 561 (49.08) | 409 (31.98) | 301 (28.24) | 294 (39.36) |
| 70-79 (n, %) | 213 (44.28) | 509 (32.52) | 382 (41.52) | 424 (33.02) | 367 (32.19) | 314 (37.56) | 648 (33.98) | 634 (40.38) | 350 (30.62) | 442 (34.56) | 499 (46.81) | 277 (37.08) |
| 80+ (n, %) | 122 (25.36) | 378 (24.15) | 156 (16.96) | 215 (16.74) | 237 (20.79) | 138 (16.51) | 338 (17.72) | 339 (21.59) | 199 (17.41) | 395 (30.88) | 255 (23.92) | 160 (21.42) |
| **Global Impression** |  |  |  |  |  |  |  |  |  |  |  |  |
| Healthy (n, %) | 24 (4.97) | 88 (5.62) | 41 (4.45) | 139 (10.83) | 61 (5.34) | 51 (6.1) | 193 (10.1) | 44 (2.8) | 48 (4.2) | 52 (4.06) | 103 (9.66) | 102 (13.53) |
| Chronic asymptomatic (n, %) | 119 (24.64) | 447 (28.53) | 234 (25.41) | 402 (31.31) | 363 (31.76) | 214 (25.6) | 906 (47.41) | 512 (32.61) | 233 (20.37) | 363 (28.36) | 298 (27.95) | 270 (35.81) |
| Chronic symptomatic (n, %) | 214 (44.31) | 647 (41.29) | 455 (49.4) | 520 (40.5) | 477 (41.73) | 401 (47.97) | 470 (24.59) | 495 (31.53) | 504 (44.06) | 367 (28.67) | 462 (43.34) | 278 (36.87) |
| Long course of decline (n, %) | 50 (10.35) | 217 (13.85) | 73 (7.93) | 100 (7.79) | 112 (9.8) | 44 (5.26) | 254 (13.29) | 306 (19.49) | 145 (12.67) | 382 (29.84) | 77 (7.22) | 49 (6.5) |
| Limited reserve with serious exacerbation (n, %) | 68 (14.08) | 164 (10.47) | 110 (11.94) | 119 (9.27) | 128 (11.2) | 123 (14.71) | 0 (0) | 213 (13.57) | 210 (18.36) | 114 (8.91) | 124 (11.63) | 51 (6.76) |
| Unknown (n, %) | 8 (1.66) | 4 (0.26) | 8 (0.87) | 4 (0.31) | 2 (0.17) | 3 (0.36) | 14 (0.73) | 0 (0) | 4 (0.35) | 2 (0.16) | 2 (0.19) | 4 (0.53) |
| **Complicating Factors** |  |  |  |  |  |  |  |  |  |  |  |  |
| Not complicated (n, %) | 96 (19.88) | 142 (9.06) | 197 (21.39) | 300 (23.36) | 143 (12.51) | 140 (16.75) | 137 (7.17) | 101 (6.43) | 51 (4.46) | 42 (3.28) | 146 (13.7) | 106 (14.06) |
| Complicated (n, %) | 383 (79.3) | 1422 (90.75) | 719 (78.07) | 983 (76.56) | 999 (87.4) | 694 (83.01) | 1772 (92.73) | 1469 (93.57) | 1092 (95.45) | 1236 (96.56) | 920 (86.3) | 646 (85.68) |
| Unknown (n, %) | 4 (0.83) | 3 (0.19) | 5 (0.54) | 1 (0.08) | 1 (0.09) | 2 (0.24) | 2 (0.1) | 0 (0) | 1 (0.09) | 2 (0.16) | 0 (0) | 2 (0.27) |
| **Health State** |  |  |  |  |  |  |  |  |  |  |  |  |
| Healthy, not complicated (n, %) | 3 (0.62) | 3 (0.19) | 13 (1.41) | 37 (2.88) | 7 (0.61) | 9 (1.08) | 13 (0.68) | 3 (0.19) | 2 (0.17) | 3 (0.23) | 10 (0.94) | 14 (1.86) |
| Healthy, complicated (n, %) | 21 (4.35) | 85 (5.42) | 28 (3.04) | 102 (7.94) | 54 (4.72) | 42 (5.02) | 180 (9.42) | 41 (2.61) | 46 (4.02) | 49 (3.83) | 93 (8.72) | 88 (11.67) |
| Chronic asymptomatic, not complicated (n, %) | 32 (6.63) | 47 (3) | 63 (6.84) | 104 (8.1) | 55 (4.81) | 35 (4.19) | 56 (2.93) | 30 (1.91) | 11 (0.96) | 10 (0.78) | 47 (4.41) | 36 (4.77) |
| Chronic asymptomatic, complicated (n, %) | 87 (18.01) | 400 (25.53) | 171 (18.57) | 298 (23.21) | 308 (26.95) | 179 (21.41) | 850 (44.48) | 482 (30.7) | 222 (19.41) | 353 (27.58) | 251 (23.55) | 234 (31.03) |
| Chronic symptomatic, not complicated (n, %) | 51 (10.56) | 76 (4.85) | 107 (11.62) | 131 (10.2) | 63 (5.51) | 78 (9.33) | 54 (2.83) | 43 (2.74) | 28 (2.45) | 10 (0.78) | 76 (7.13) | 45 (5.97) |
| Chronic symptomatic, complicated (n, %) | 163 (33.75) | 571 (36.44) | 348 (37.79) | 389 (30.3) | 414 (36.22) | 323 (38.64) | 416 (21.77) | 452 (28.79) | 476 (41.61) | 357 (27.89) | 386 (36.21) | 233 (30.9) |
| Long course of decline, not complicated (n, %) | 6 (1.24) | 11 (0.7) | 6 (0.65) | 17 (1.32) | 10 (0.87) | 8 (0.96) | 13 (0.68) | 18 (1.15) | 5 (0.44) | 16 (1.25) | 7 (0.66) | 8 (1.06) |
| Long course of decline, complicated (n, %) | 44 (9.11) | 206 (13.15) | 67 (7.27) | 83 (6.46) | 102 (8.92) | 36 (4.31) | 241 (12.61) | 288 (18.34) | 140 (12.24) | 366 (28.59) | 70 (6.57) | 41 (5.44) |
| Limited reserve with serious exacerbation, not complicated (n, %) | 3 (0.62) | 5 (0.32) | 8 (0.87) | 11 (0.86) | 8 (0.7) | 10 (1.2) | 0 (0) | 7 (0.45) | 5 (0.44) | 3 (0.23) | 6 (0.56) | 2 (0.27) |
| Limited reserve with serious exacerbation, complicated (n, %) | 65 (13.46) | 159 (10.15) | 102 (11.07) | 108 (8.41) | 120 (10.5) | 113 (13.52) | 74 (3.87) | 206 (13.12) | 205 (17.92) | 111 (8.67) | 118 (11.07) | 49 (6.5) |
| Unknown (n, %) | 8 (1.66) | 4 (0.26) | 8 (0.87) | 4 (0.31) | 2 (0.17) | 3 (0.36) | 14 (0.73) | 0 (0) | 4 (0.35) | 2 (0.16) | 2 (0.19) | 4 (0.53) |
