# Supplementary material for: Validation and application of a needs‐based segmentation tool for cross‐country comparisons
Source: Health Serv Res. 2021 Nov 10;56(Suppl 3):1394–404. doi: 10.1111/1475-6773.13873 (PMC8579203; doi:10.1111/1475-6773.13873)

**Figure 1A: Kaplan-Meier survival estimates for mortality by GI segment (Austria, Germany, Sweden, Netherlands)**


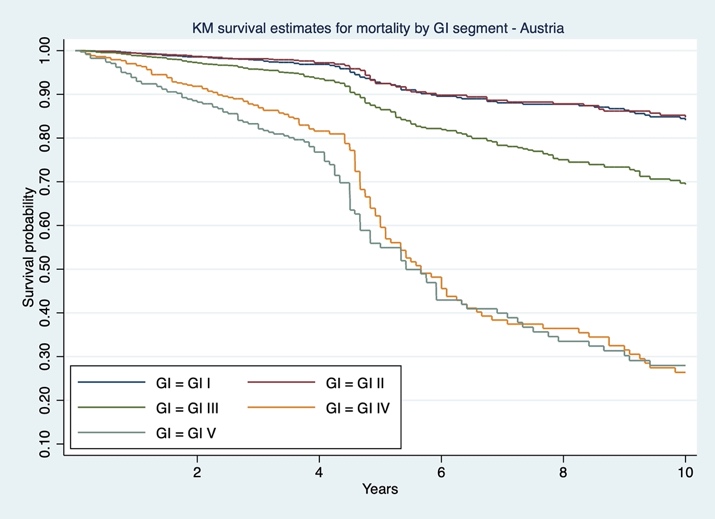

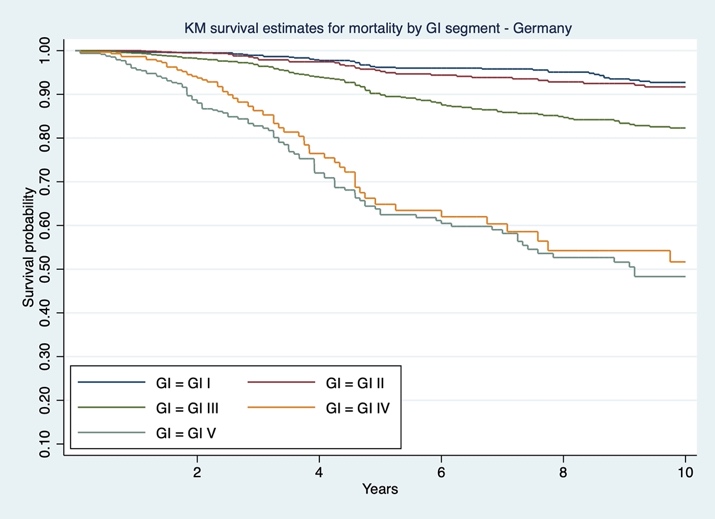

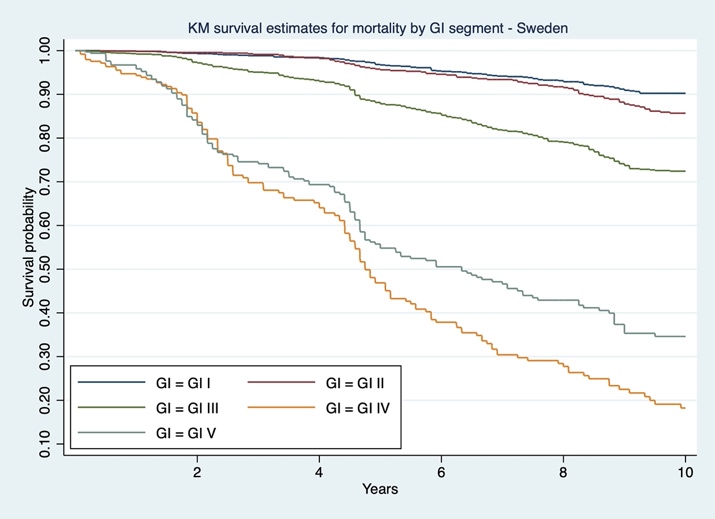

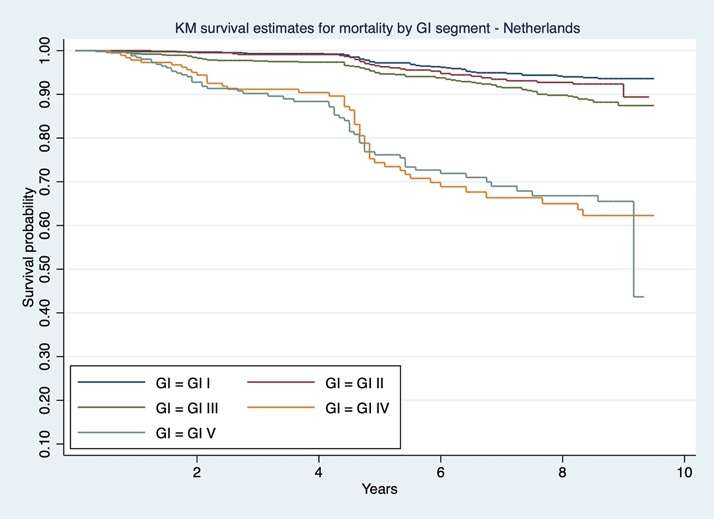


**Figure 1B: Kaplan-Meier survival estimates for mortality by GI segment (Spain, Italy, France, Denmark)**


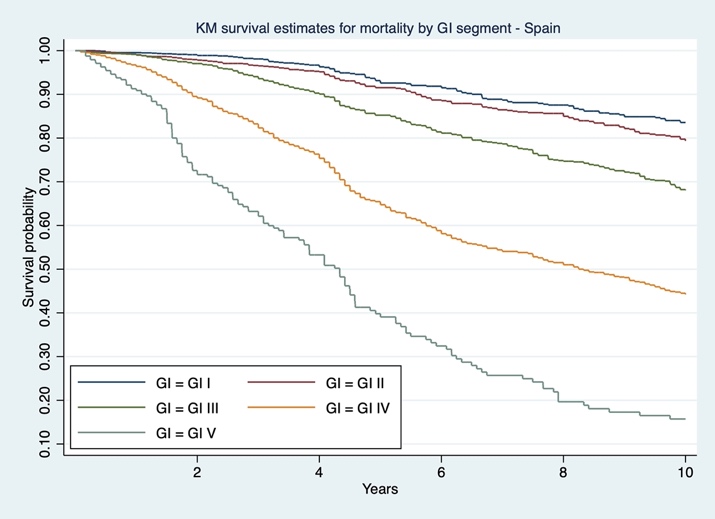

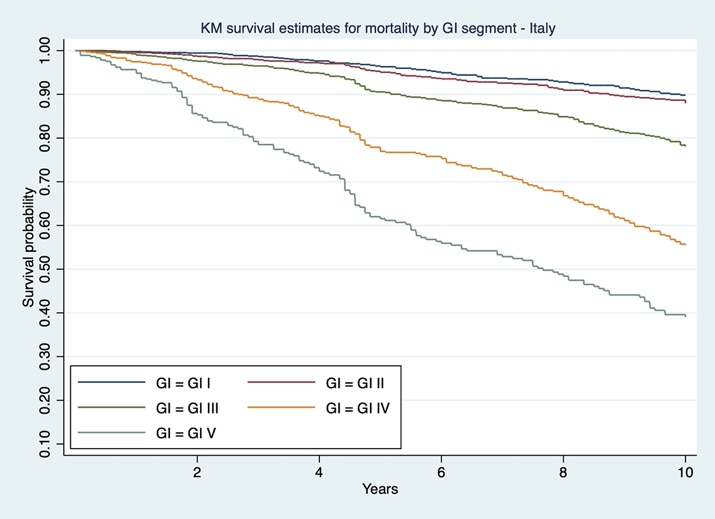

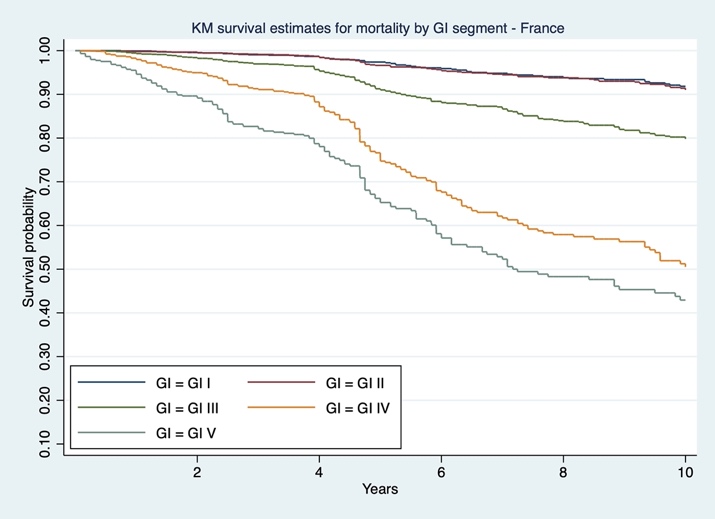

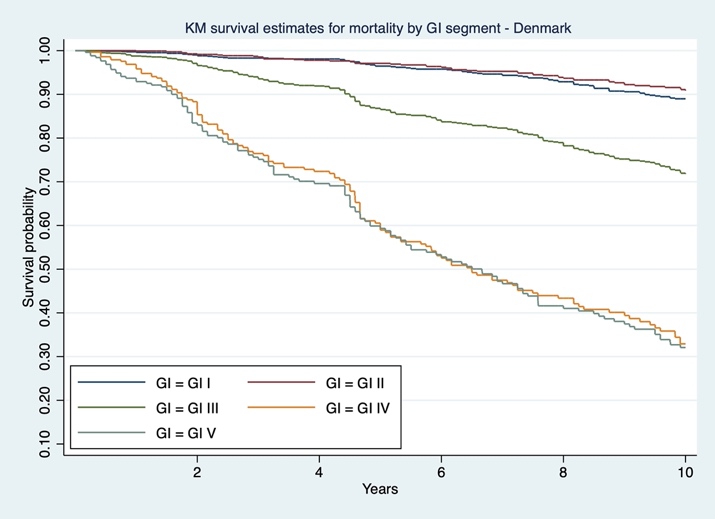


**Figure 1C: Kaplan-Meier survival estimates for mortality by GI segment (Greece, Switzerland, Belgium, Israel)**


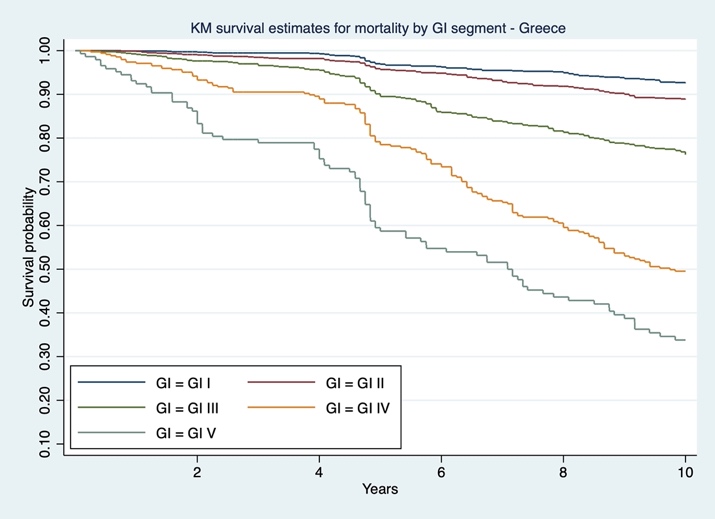

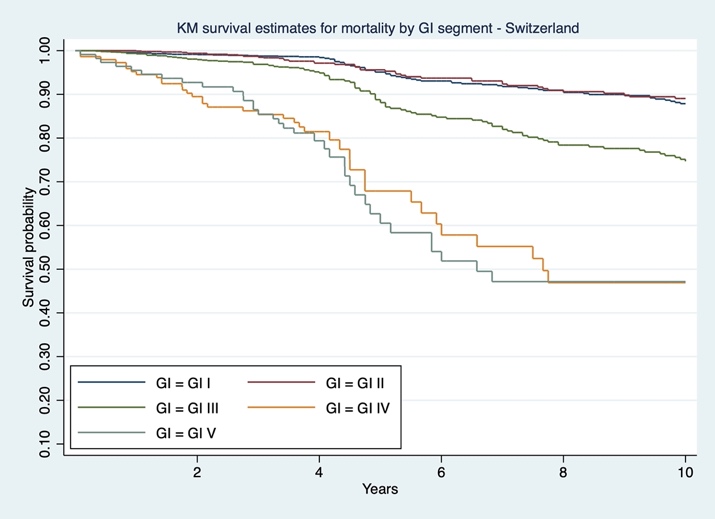

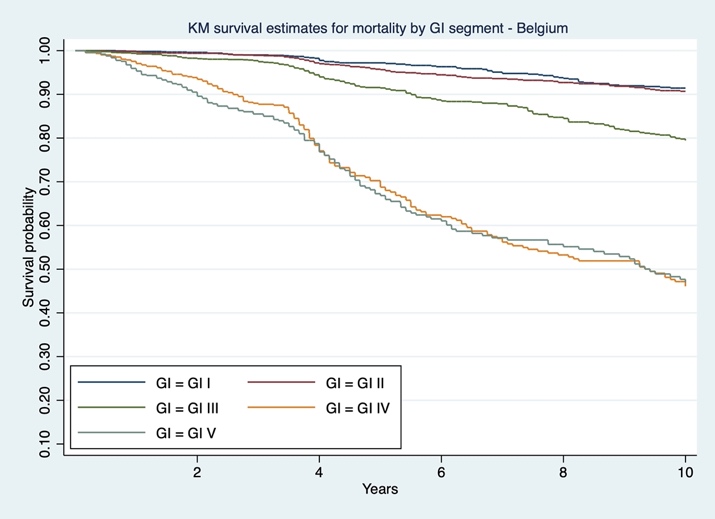

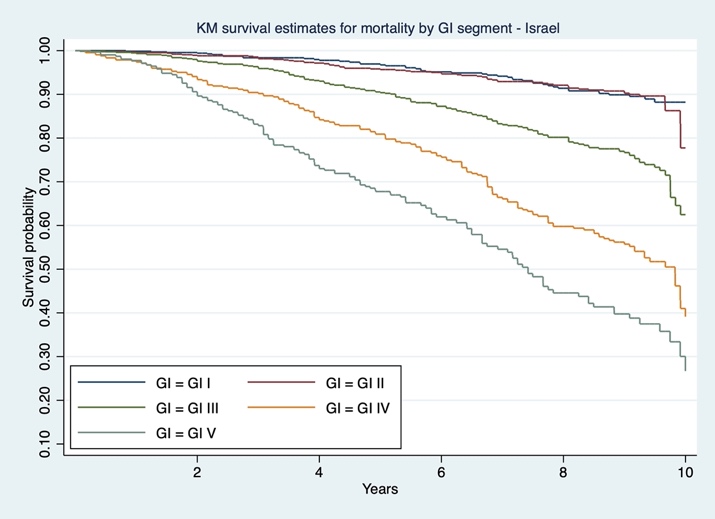


**Figure 1D: Kaplan-Meier survival estimates for mortality by GI segment (Czech Republic, Poland)**


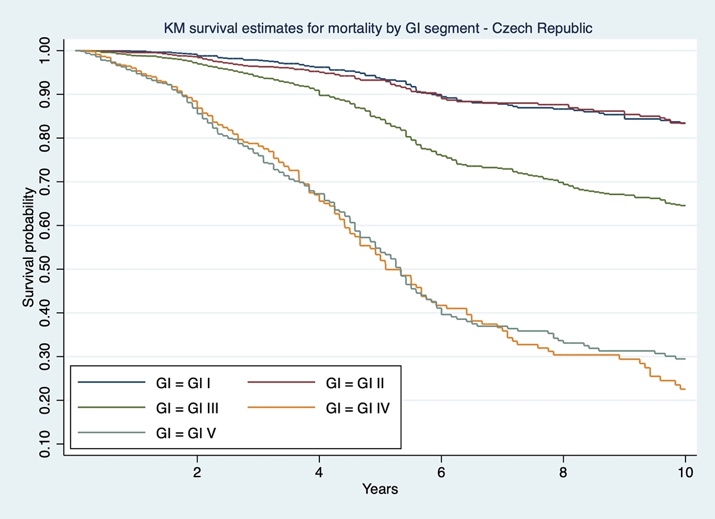

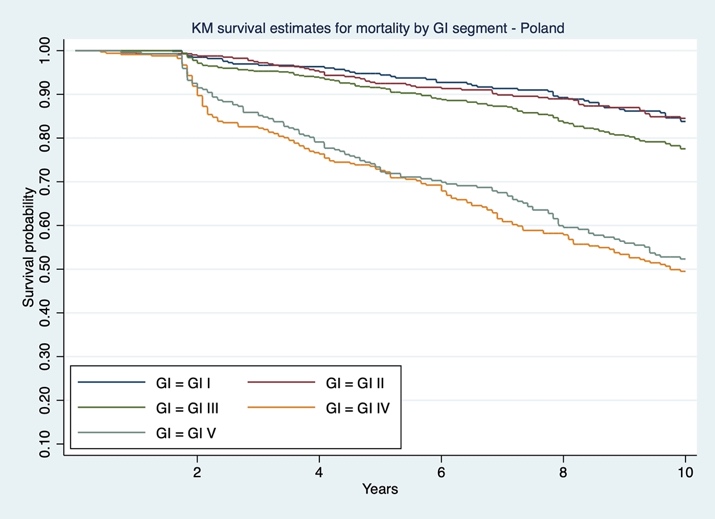


**Figure 2A: Kaplan-Meier survival estimates for mortality by CF segment (Austria, Germany, Sweden, Netherlands)**


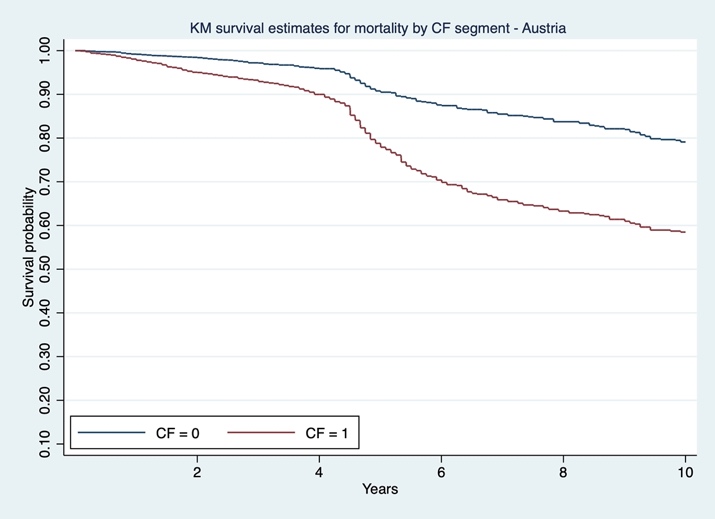

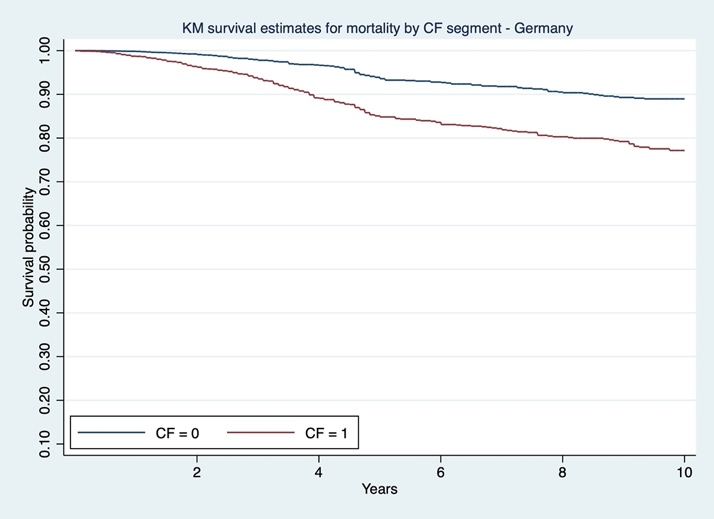

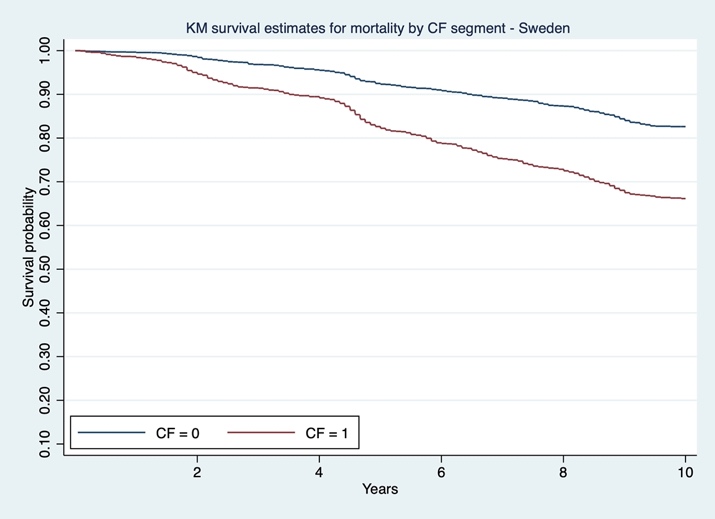

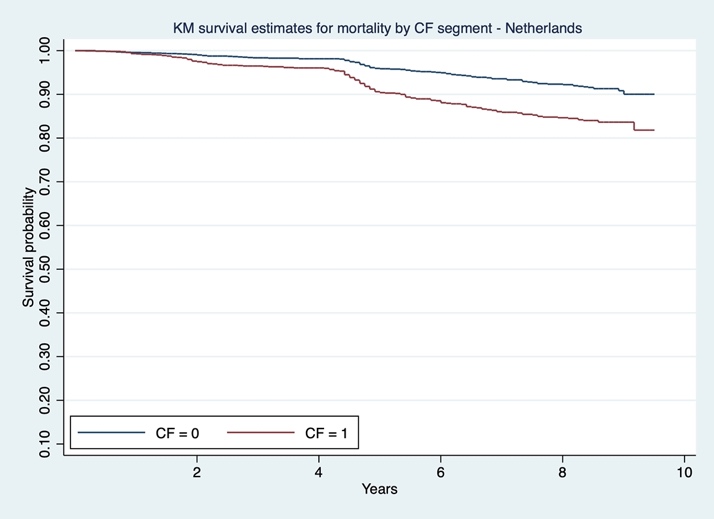


**Figure 2B: Kaplan-Meier survival estimates for mortality by CF segment (Spain, Italy, France, Denmark)**


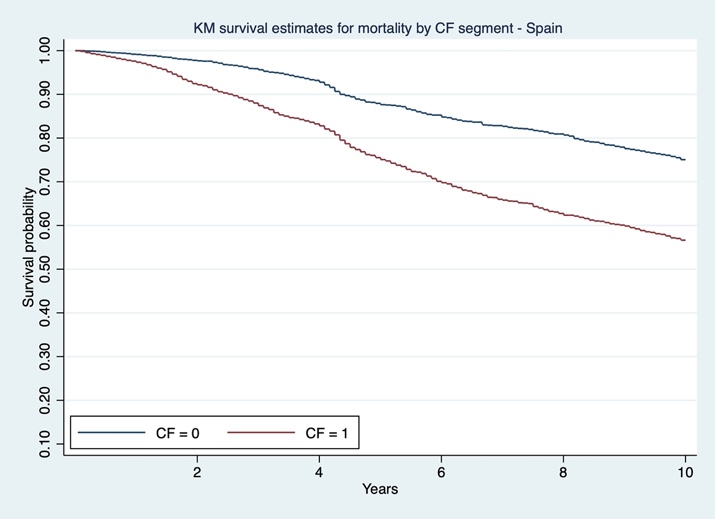

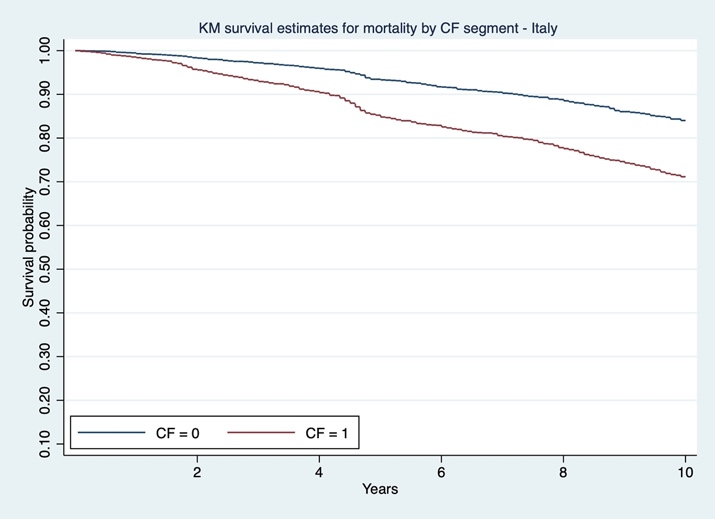

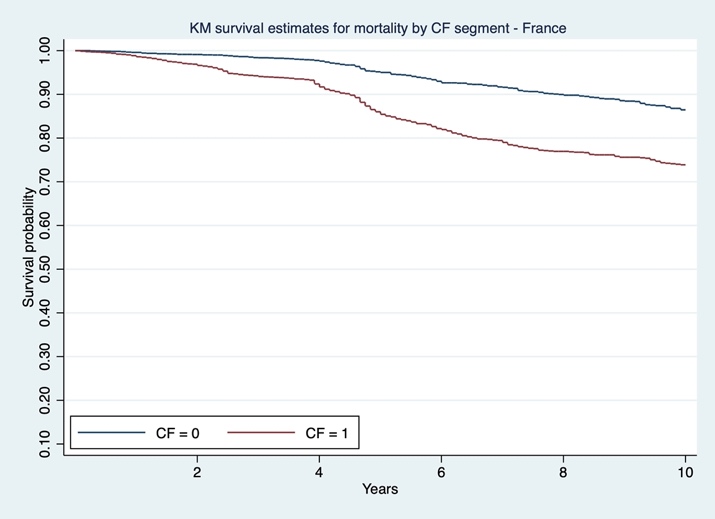

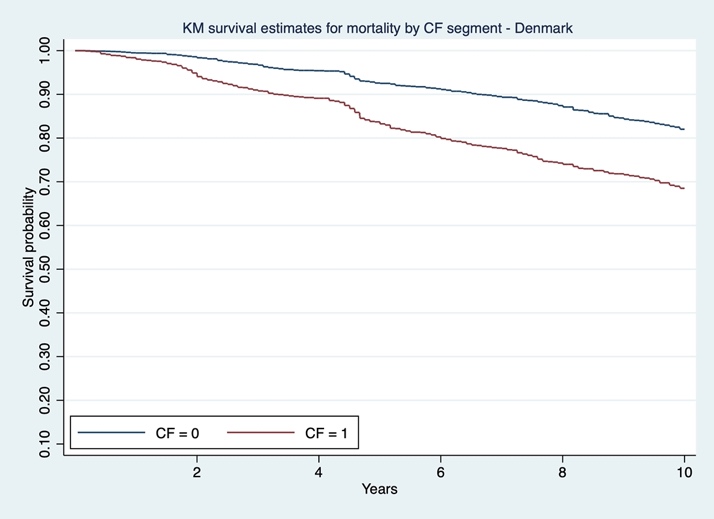


**Figure 2C: Kaplan-Meier survival estimates for mortality by CF segment (Greece, Switzerland, Belgium, Israel)**


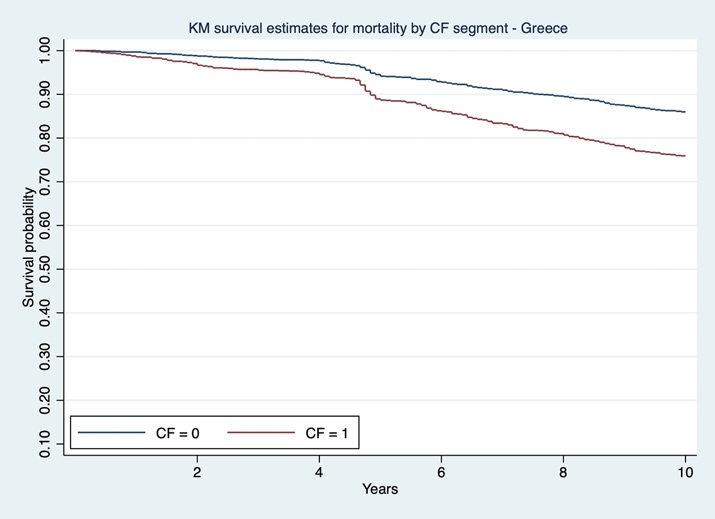

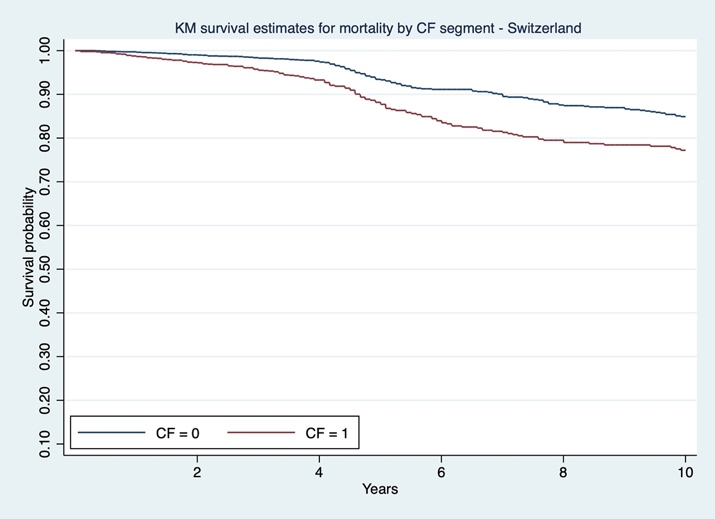

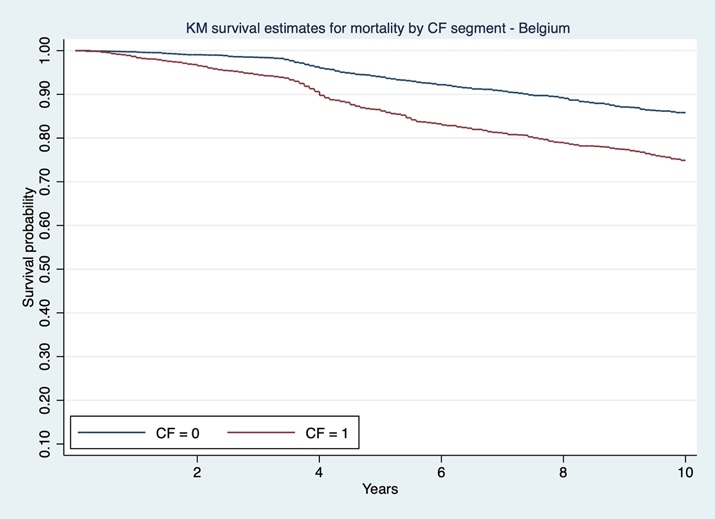

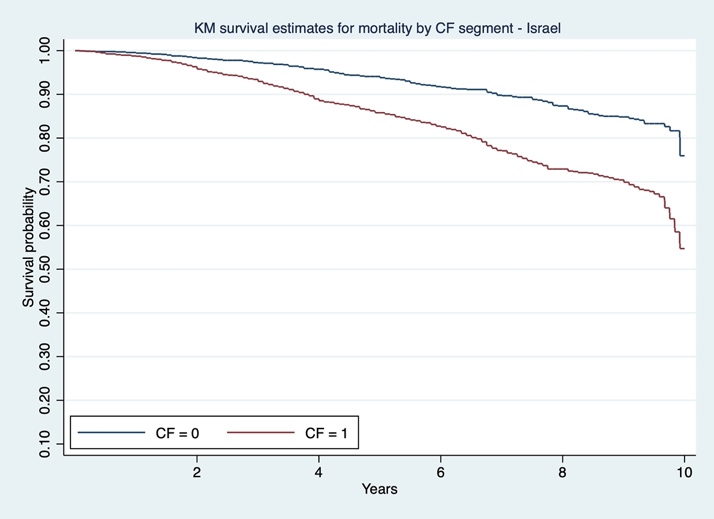


**Figure 2D: Kaplan-Meier survival estimates for mortality by CF segment (Czech Republic, Poland)**


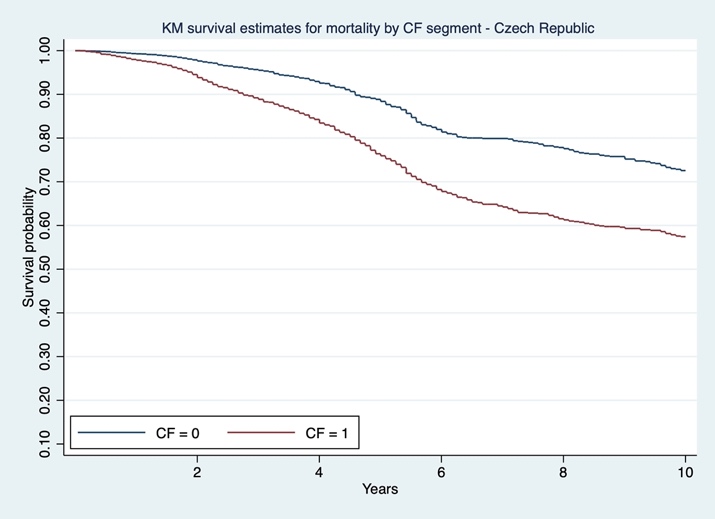

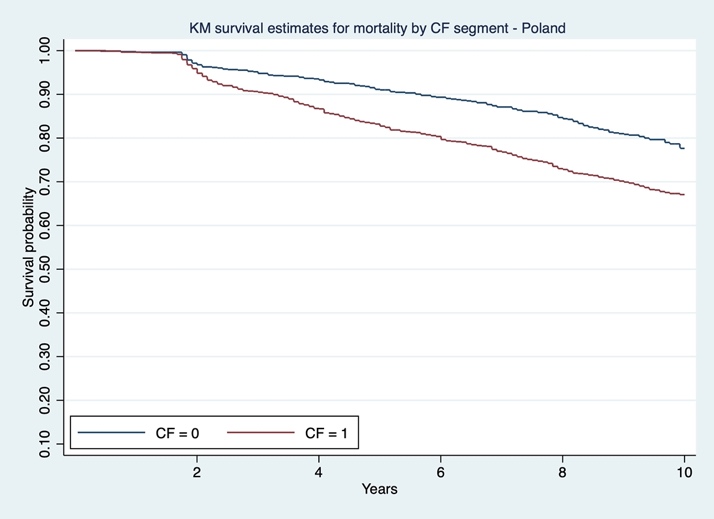

Supplement: Supplementary file 3 — Data S3. Supporting information. [file HESR-56-1394-s002.docx]
