# Supplementary material for: Validation and application of a needs‐based segmentation tool for cross‐country comparisons
Source: Health Serv Res. 2021 Nov 10;56(Suppl 3):1394–404. doi: 10.1111/1475-6773.13873 (PMC8579203; doi:10.1111/1475-6773.13873)
Supplement: Supplementary file 4 — Data S4. Supporting information. [file HESR-56-1394-s004.docx]

Table 1: Weighted CCSST-assigned Global Impressions (GI) segments with and without Complicating Factors (CFs) per country in percentage. Data collected in 2015 for all countries except The Netherlands, for which we use data from 2013.

|  | **Country** | | | | | | | | | | | | | |
| --- | --- | --- | --- | --- | --- | --- | --- | --- | --- | --- | --- | --- | --- | --- |
| **Characteristics** | **Austria** | **Belgium** | **Czech Republic** | **Denmark** | **France** | **Germany** | **Greece** | **Israel** | **Italy** | **Netherlands** | **Poland** | **Spain** | **Sweden** | **Switzerland** |
| **Gender** |  |  |  |  |  |  |  |  |  |  |  |  |  |  |
| Female (%) | 53.99 | 53.43 | 54.60 | 52.29 | 54.50 | 53.62 | 54.02 | 53.69 | 54.41 | 52.41 | 55.99 | 53.83 | 52.12 | 52.87 |
| Male (%) | 46.01 | 46.57 | 45.40 | 47.71 | 45.50 | 46.38 | 45.98 | 46.31 | 45.59 | 47.59 | 44.01 | 46.17 | 47.88 | 47.13 |
| **Age** |  |  |  |  |  |  |  |  |  |  |  |  |  |  |
| 50-59 (%) | 35.20 | 35.94 | 33.72 | 33.42 | 34.30 | 34.67 | 31.72 | 34.10 | 32.65 | 37.32 | 38.31 | 35.89 | 31.58 | 35.93 |
| 60-69 (%) | 28.82 | 29.52 | 35.06 | 33.40 | 30.81 | 26.90 | 28.53 | 35.75 | 28.13 | 31.70 | 32.45 | 27.16 | 32.33 | 30.18 |
| 70-79 (%) | 22.12 | 19.69 | 20.11 | 21.46 | 19.16 | 25.02 | 22.86 | 17.63 | 22.91 | 19.27 | 17.98 | 20.49 | 21.89 | 19.97 |
| 80+ (%) | 13.86 | 14.85 | 11.11 | 11.72 | 15.74 | 13.41 | 16.89 | 12.53 | 16.31 | 11.71 | 11.25 | 16.46 | 14.19 | 13.92 |
| **Global Impression** |  |  |  |  |  |  |  |  |  |  |  |  |  |  |
| Healthy (%) | 14.32 | 12.23 | 11.80 | 19.29 | 12.63 | 14.31 | 21.29 | 18.97 | 17.77 | 27.70 | 14.34 | 12.49 | 21.21 | 21.07 |
| Chronic asymptomatic (%) | 26.24 | 28.85 | 28.32 | 28.63 | 30.93 | 22.24 | 34.47 | 28.74 | 30.24 | 19.05 | 22.55 | 37.47 | 28.16 | 34.11 |
| Chronic symptomatic (%) | 36.18 | 32.63 | 37.70 | 35.68 | 31.25 | 41.24 | 21.36 | 23.45 | 22.85 | 34.64 | 30.66 | 24.34 | 32.14 | 31.60 |
| Long course of decline (%) | 11.25 | 16.59 | 11.13 | 10.03 | 15.07 | 10.23 | 16.64 | 15.84 | 18.33 | 7.61 | 17.94 | 21.43 | 10.26 | 8.33 |
| Limited reserve with serious exacerbation (%) | 9.53 | 9.09 | 10.71 | 6.02 | 9.29 | 11.62 | 4.98 | 10.81 | 9.38 | 10.17 | 13.44 | 3.99 | 7.66 | 4.60 |
| Unknown (%) | 2.47 | 0.62 | 0.34 | 0.35 | 0.81 | 0.36 | 1.25 | 2.19 | 1.42 | 0.83 | 1.07 | 0.28 | 0.57 | 0.29 |
| **Complicating Factors** |  |  |  |  |  |  |  |  |  |  |  |  |  |  |
| Not complicated (%) | 55.05 | 52.42 | 52.59 | 63.73 | 54.11 | 59.39 | 53.14 | 52.97 | 55.81 | 61.14 | 52.59 | 50.08 | 62.86 | 64.07 |
| Complicated (%) | 44.82 | 47.45 | 47.34 | 36.21 | 45.63 | 40.50 | 46.84 | 46.81 | 43.96 | 38.76 | 47.02 | 49.85 | 36.97 | 35.90 |
| Unknown (%) | 0.13 | 0.13 | 0.07 | 0.06 | 0.27 | 0.11 | 0.02 | 0.22 | 0.23 | 0.10 | 0.40 | 0.06 | 0.16 | 0.03 |
| **Health State** |  |  |  |  |  |  |  |  |  |  |  |  |  |  |
| Healthy, not complicated (%) | 10.73 | 9.12 | 7.97 | 14.32 | 9.66 | 10.86 | 14.33 | 13.09 | 12.99 | 20.71 | 10.36 | 7.36 | 15.86 | 15.41 |
| Healthy, complicated (%) | 3.59 | 3.11 | 3.82 | 4.97 | 2.97 | 3.45 | 6.97 | 5.88 | 4.78 | 6.99 | 3.98 | 5.13 | 5.34 | 5.66 |
| Chronic asymptomatic, not complicated (%) | 17.78 | 20.48 | 19.55 | 21.76 | 21.39 | 16.15 | 22.20 | 19.80 | 20.62 | 13.29 | 15.40 | 25.20 | 21.66 | 24.69 |
| Chronic asymptomatic, complicated (%) | 8.46 | 8.36 | 8.77 | 6.87 | 9.54 | 6.09 | 12.27 | 8.94 | 9.61 | 5.76 | 7.15 | 12.27 | 6.50 | 9.42 |
| Chronic symptomatic, not complicated (%) | 22.59 | 19.38 | 21.87 | 24.53 | 19.35 | 27.92 | 11.30 | 13.27 | 14.17 | 22.21 | 19.45 | 12.27 | 21.22 | 21.21 |
| Chronic symptomatic, complicated (%) | 13.59 | 13.25 | 15.83 | 11.14 | 11.91 | 13.32 | 10.06 | 10.18 | 8.68 | 12.43 | 11.22 | 12.07 | 10.93 | 10.40 |
| Long course of decline, not complicated (%) | 2.11 | 2.23 | 1.39 | 2.01 | 2.51 | 1.86 | 4.24 | 4.61 | 6.42 | 1.71 | 4.39 | 5.01 | 2.16 | 1.68 |
| Long course of decline, complicated (%) | 9.14 | 14.36 | 9.74 | 8.02 | 12.56 | 8.36 | 12.40 | 11.23 | 11.91 | 5.91 | 13.55 | 16.42 | 8.10 | 6.65 |
| Limited reserve with serious exacerbation, not complicated (%) | 0.98 | 0.96 | 1.74 | 0.96 | 1.04 | 2.48 | 0.31 | 1.10 | 1.17 | 3.06 | 2.71 | 0.22 | 1.74 | 1.02 |
| Limited reserve with serious exacerbation, complicated (%) | 8.55 | 8.14 | 8.97 | 5.07 | 8.26 | 9.14 | 4.67 | 9.71 | 8.21 | 7.11 | 10.73 | 3.77 | 5.92 | 3.57 |
| Unknown (%) | 2.47 | 0.62 | 0.34 | 0.35 | 0.81 | 0.36 | 1.25 | 2.19 | 1.42 | 0.83 | 1.07 | 0.28 | 0.57 | 0.29 |
