# Supplementary material for: Validation and application of a needs‐based segmentation tool for cross‐country comparisons
Source: Health Serv Res. 2021 Nov 10;56(Suppl 3):1394–404. doi: 10.1111/1475-6773.13873 (PMC8579203; doi:10.1111/1475-6773.13873)
Supplement: Supplementary file 5 — Data S5. Supporting information. [file HESR-56-1394-s003.docx]

Appendix E: Transition probability estimates

Table 1: Transition intensities

| **Transition intensities** | | | | |
| --- | --- | --- | --- | --- |
|  |  | **To** | | |
|  |  | **State 1** | **State 2** | **State 3** |
| **From** | **State 1** | -0.0722190 [-0.0737932,-0.070678] | 0.0715671 [ 0.0699810, 0.073189] | 0.0006519 [ 0.0003821, 0.001112] |
|  | **State 2** | 0.1994133 [ 0.1935292, 0.205476] | -0.2621634 [-0.2690056,-0.255495] | 0.0627501 [ 0.0596238, 0.066040] |
|  | **State 3** | 0 | 0 | 0 |

Table 2: Hazard ratios

|  | **Hazard ratios** | | | | | | |
| --- | --- | --- | --- | --- | --- | --- | --- |
|  | **State 1 - State 2** |  | **State 1 - State 3** |  | **State 2 - State 1** |  | **State 2 - State 3** |
|  |  |  |  |  |  |  |  |
| **Gender** |  |  |  |  |  |  |  |
| Female | 1.1160 [1.0754, 1.1582] |  | 0.2396 [0.1390, 0.4128] |  | 1.1056 [1.0475, 1.1668] |  | 0.6506 [0.6224, 0.6800] |
| Male (Baseline) | 1 |  | 1 |  | 1 |  | 1 |
| **Presence of any CFs** |  |  |  |  |  |  |  |
| With CFs | 1 |  | 1 |  | 1 |  | 1 |
| No CFs | 0.7275 [0.7006, 0.7555] |  | 1.2934 [0.8387, 1.9944] |  | 1.9020 [1.7977, 2.0123] |  | 0.9273 [0.8768, 0.9806] |
| **Age** |  |  |  |  |  |  |  |
| 50 - 59 (Baseline) | 1 |  | 1 |  | 1 |  | 1 |
| 60-69 | 1.6210 [1.5241, 1.7241] |  | 1.3315 [0.8824, 2.0092] |  | 0.8698 [0.8081, 0.9362] |  | 1.6077 [1.4044, 1.8404] |
| 70-79 | 3.6566 [3.44444, 3.8819] |  | 0.4243 [0.07913, 2.2751] |  | 0.5938 [0.55141, 0.6395] |  | 2.2229 [1.94883, 2.5355] |
| 80+ | 8.2905 [7.78019, 8.8342] |  | 0.1521 [0.02145, 1.0793] |  | 0.3603 [0.32898, 0.3945] |  | 3.6644 [3.22779, 4.1601] |
| **Country** |  |  |  |  |  |  |  |
| Austria | 0.9542 [0.8731, 1.043] |  | 1.7804 [0.8204, 3.864] |  | 1.0803 [0.9513, 1.227] |  | 1.2019 [1.0711, 1.349] |
| Belgium (Baseline) | 1 |  | 1 |  | 1 |  | 1 |
| Czech Republic | 1.147 [1.0561, 1.245] |  | 2.512 [1.2595, 5.008] |  | 1.081 [0.9609, 1.215] |  | 1.584 [1.4296, 1.755] |
| Denmark | 0.7066 [0.6454, 0.7735] |  | 0.7674 [0.2505, 2.3508] |  | 0.7093 [0.6126, 0.8212] |  | 1.7462 [1.5665, 1.9464] |
| France | 1.0105 [0.9314, 1.096] |  | 1.2687 [0.5890, 2.733] |  | 0.9590 [0.8542, 1.077] |  | 0.9081 [0.8139, 1.013] |
| Germany | 0.8359 [0.7619, 0.9171] |  | 0.7499 [0.2631, 2.1375] |  | 1.0154 [0.8921, 1.1557] |  | 1.0666 [0.9364, 1.2149] |
| Greece | 0.8013 [0.7230, 0.8880] |  | 0.8280 [0.3178, 2.1570] |  | 0.7394 [0.6237, 0.8765] |  | 1.3388 [1.2025, 1.4907] |
| Israel | 0.9641 [0.8744, 1.0631] |  | 1.2137 [0.4280, 3.4419] |  | 0.5196 [0.4485, 0.6019] |  | 0.9899 [0.8847, 1.1076] |
| Italy | 1.4096 [1.3029, 1.525] |  | 0.9934 [0.3931, 2.510] |  | 0.8992 [0.8042, 1.005] |  | 0.9548 [0.8602, 1.060] |
| Netherlands | 0.8281 [0.7456, 0.9197] |  | 1.3302 [0.5180, 3.4156] |  | 0.7939 [0.6811, 0.9254] |  | 1.0887 [0.9278, 1.2776] |
| Poland | 1.505 [1.3472, 1.6810] |  | 4.869 [2.4423, 9.7088] |  | 0.770 [0.6651, 0.8915] |  | 1.157 [1.0236, 1.3078] |
| Spain | 1.552 [1.4377, 1.675] |  | 1.585 [0.7316, 3.434] |  | 1.123 [1.0123, 1.246] |  | 1.155 [1.0534, 1.267] |
| Sweden | 0.7324 [0.6728, 0.7974] |  | 0.5105 [0.1084, 2.4033] |  | 0.8033 [0.6974, 0.9253] |  | 1.5112 [1.3602, 1.6789] |
| Switzerland | 0.6433 [0.57916, 0.7146] |  | 0.4473 [0.09836, 2.0341] |  | 1.3996 [1.19929, 1.6335] |  | 1.4464 [1.25452, 1.6676] |
